# Diversity and Spatiotemporal Distribution of Fish in a Highland Lake in China Based on Environmental DNA Metabarcoding

**DOI:** 10.1002/ece3.73082

**Published:** 2026-02-11

**Authors:** Lu Shu, Arne Ludwig, Hongmei Pan, Jiayan Lin, Yuan Xu, Hang Shan, Te Cao, Zuogang Peng

**Affiliations:** ^1^ Key Laboratory of Freshwater Fish Reproduction and Development (Ministry of Education) Southwest University School of Life Sciences Chongqing China; ^2^ Department of Evolutionary Genetics Leibniz Institute for Zoo and Wildlife Research Berlin Germany; ^3^ Donghu Experimental Station of Lake Ecosystems, State Key Laboratory of Freshwater Ecology and Biotechnology Institute of Hydrobiology, Chinese Academy of Sciences Wuhan China

**Keywords:** eDNA metabarcoding, fish community structure, freshwater biodiversity, lake ecosystem, temporal variation

## Abstract

Local fish diversity in lakes has severely declined in the last century under the effects of climate change and human activities. Thus, examining the underlying factors and implementing appropriate measures are crucial for preventing further aquatic biodiversity losses. Environmental DNA (eDNA) metabarcoding represents a promising tool for improving fish population monitoring. While spatiotemporal variations of fish eDNA in lentic ecosystems have become a research focus, effective monitoring techniques remain limited. Therefore, this study used eDNA metabarcoding to monitor the diversity and spatiotemporal distribution of fish in Erhai Lake, China. Water samples from the shore, nearshore, and midline were collected from 2020 to 2021 during summer and autumn. Thirty‐six taxa, including 5 native (one endangered species, *Schizothorax taliensis*) and 31 non‐native taxa, were detected. Seasonal and spatial differences in fish community structure were observed. The seasonal distribution was primarily influenced by water temperature and nutrient status, while the spatial distribution was affected by water depth. Most fish species found in the lake were detected in shoreline samples, suggesting that shoreline sampling is a cost‐effective strategy for monitoring fish diversity. These findings confirmed that fine‐scale spatial sampling and eDNA metabarcoding represent effective tools for monitoring fish diversity and spatiotemporal distribution in lakes.

## Graphical abstract

We analyzed water samples from different seasons and locations in Erhai Lake to determine whether eDNA barcoding can effectively show the diversity of fish in such lakes. In the manuscript, we discuss our findings regarding the range of species found, their spatiotemporal distribution, and whether eDNA barcoding gave similar results to traditional diversity surveying.

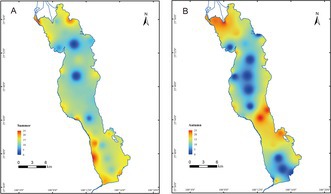

## 1 Introduction

Freshwater lakes support rich fish biodiversity, which is essential for ecosystem functioning and local fisheries (Levêque et al. 2008). However, many of these fish populations are rapidly declining due to increasing human pressures (Tickner et al. 2020). Erhai Lake (25°36′–25°58′ N, 100°05′–100°17′ E), located in Yunnan Province, China, is an iconic high‐altitude plateau lake (~1974 m above sea level) with an area of approximately 256 km^2^ (~40 km long and 7–8 km wide) (Wang et al. 2022). Historically a biodiversity hotspot, Erhai's fish community has undergone significant changes over the past few decades (Fei et al. 2011). Nutrient pollution, overfishing, and the introduction of non‐native species have altered the community composition. Notably, Erhai's indigenous fish species have experienced a sharp decline, with most of the seven endemic species now extirpated (only two remain). In contrast, 22 introduced species now dominate the fish community (Tang et al. 2013). These changes illustrate the vulnerability of lentic ecosystems, with Erhai experiencing significant ecological and fishery changes since the 1960s (Du and Li 2001; Yan et al. 2012). This underscores the urgent need for effective biodiversity monitoring in Erhai and similar lakes.

Traditional fish survey methods, such as electrofishing, gill‐netting, seine netting, trap nets, and hydroacoustic surveys, have long been employed to inventory fish populations (Bayley and Peterson 2001). However, these techniques are labor‐intensive, ecologically disruptive, time‐consuming, and often suffer from various biases, such as low sensitivity and incomplete species detection. As a result, environmental DNA (eDNA) metabarcoding has emerged as a promising alternative (Miya et al. 2015; Valentini et al. 2016). This non‐invasive method involves extracting DNA from water samples, followed by polymerase chain reaction (PCR) amplification using universal primers, and high‐throughput sequencing (Bohmann et al. 2014). eDNA metabarcoding offers a more efficient sampling approach while significantly improving species detection sensitivity compared to traditional methods (Ruppert et al. 2019).

The value of eDNA metabarcoding for characterizing fish communities is well established and offers strong potential for aquatic biodiversity assessment (Jerde et al. 2013; Keskin et al. 2016; Li et al. 2019). Intensive spatial and seasonal sampling can improve species detection and abundance inference in large lentic systems (Hänfling et al. 2016; Handley et al. 2019; Rund et al. 2025; Sellers et al. 2024), yet many studies still lack adequate temporal replication to resolve fine‐scale patterns. In Erhai, Shu et al. (2020) provided an initial snapshot but highlighted the need for broader coverage. To date, no study has combined repeated intra‐seasonal replicates with o offshore sampling and integrated water‐column sampling at deep sites. This is important because seasonal thermal stratification can retain eDNA below the thermocline, leading surface samples to under‐detect deep‐water taxa (Handley et al. 2019). Accordingly, offshore sampling coupled with integrated water‐column sampling at deep sites can complement shoreline surveys and reveal taxa that would otherwise be missed.

In this study, we address these gaps by conducting an expanded seasonal eDNA survey of Erhai Lake. We conducted sampling in the summer and autumn of 2020 and 2021, with technical replicates at each site, including both shoreline and offshore (nearshore and midline) stations across the lake's basins. Our primary objectives are twofold: (1) to enhance the accuracy and completeness of species detection through replicated eDNA metabarcoding, and (2) to characterize the spatiotemporal distribution and community dynamics of fish populations in Erhai. By integrating high‐resolution eDNA data with temporal and spatial replication, we aim to generate a spatially and seasonally resolved characterization of fish diversity in the lake, including species richness, community composition, and their distribution patterns. Such information is essential for guiding conservation and management efforts: as a critical drinking water source and biodiversity refuge, Erhai's recovery depends on reliable monitoring. Therefore, this study thus serves as a case study of advanced eDNA monitoring in large lakes, providing crucial data to support ecological conservation policies for Erhai and similar lentic ecosystems.

## 2 Materials and Methods

### 2.1 Study Site and Sample Collection

**FIGURE 1:**
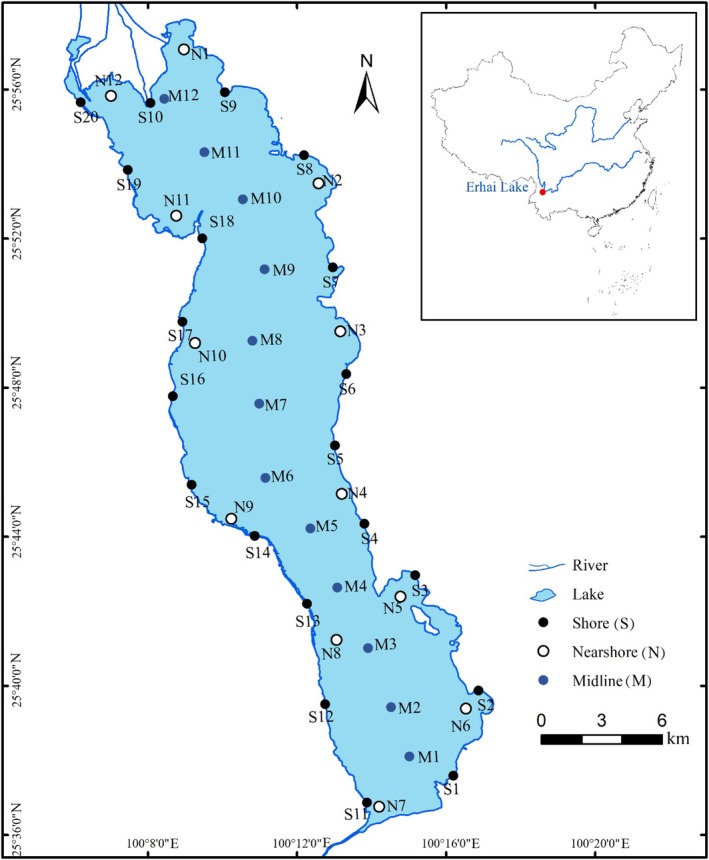
Sampling sites in Erhai Lake. Sampling overview across four campaigns. We collected 132 water samples per campaign (total = 528). Of these, 355 samples (summer 2020: *N* = 132, autumn 2020: *N* = 52, summer 2021: *N* = 131, autumn 2021: *N* = 40) yielded successful amplification and were analyzed. See Methods for details on PCR success and sequencing inclusion criteria.

To assess the temporal and spatial distribution of fish diversity, this study conducted four sampling events during summer (June 2020 and 2021) and autumn (November 2020 and September 2021) at fine spatial scales. Summer and autumn samplings were repeated twice to ensure the reproducibility and reliability of the results. In total, 44 sites were sampled, with each sampling event including 20 shore sites, 12 nearshore sites, and 12 midline sites (Figure 1). For shoreline sampling, 1 L water samples were collected from the shore by submerging a sterile plastic bottle 5–10 cm below the surface. For offshore sampling, a boat was used to collect 1 L water samples from the upper (5 cm below the surface), middle, and lower (5 cm above the bottom) layers, which were stratified according to water depth, as measured by a portable ultrasonic depth sounder (SpeedTech, Lancaster, CA, USA). The water sampler was decontaminated in the laboratory prior to each field sampling by soaking in a 10% bleach solution, followed by rinsing with sterile water and air‐drying in a laminar flow hood (Kumar et al. 2019). The sampler was then sealed in a sterile bag for transport to the field. After sampling, individual samples from the different water layers (upper, middle, and lower) were combined and mixed thoroughly, and a 1 L aliquot was transferred into a pre‐sterilized, labeled plastic bottle. These subsamples were sealed and stored with ice packs and transported to the laboratory, where they were stored at 4°C until filtration within 6 h to minimize DNA degradation. Three replicate water samples were collected at each site, totaling 132 samples per sampling event (528 samples in total across four events).

Water samples were filtered using 0.45 μm mixed cellulose acetate and nitrate filters (Whatman, Maidstone, UK). Cross‐contamination was avoided by bleaching all equipment with a 10% bleach solution and rinsing with sterile water after every filtration for each triplicate sample. In addition, ddH_2_O (1 L) was filtered to check for contamination. Replicate samples from each site were filtered separately, resulting in a total of 176 filtration blanks across four sampling events. All filters were subsequently stored at −20°C until DNA extraction.

Additionally, environmental factors, including water temperature (T), dissolved oxygen (DO), conductivity (Con), pH, water depth (Dep), nitrate (NO_3_^−^), phosphite (PO_3_^−^), ammonium (NH_4_^+^), total nitrogen (TN), total phosphorus (TP), and chlorophyll‐a (Chl‐a), were measured following water sampling. Except for water depth, all parameters were recorded at each site using a HYDROLAB HL7 multiparameter sonde (Ott HydroMet, Loveland, CO) in accordance with standard operating procedures. These environmental variables were subsequently used in the redundancy analysis (RDA).

### 2.2 eDNA Extraction, Amplification, and Sequencing

eDNA was isolated from filters using a PowerWater DNA Isolation Kit (MoBio, Carlsbad, CA, USA) following the manufacturer's protocol. A blank filter was included to control contamination during DNA extraction, with one blank per extraction. DNA extractions were performed separately for shore, nearshore, and midline samples, totaling 12 extraction blanks across four sampling events. All eDNA extracts were stored at −20°C until PCR amplification.

The fish universal primer “Tele02” was used to amplify eDNA templates, and it was specifically designed for a 129–209 bp fragment of the highly variable region of the mitochondrial 12S rRNA gene (Taberlet et al. 2018). All PCR amplifications were performed in triplicate. eDNA extracts were added to a solution (25 μL total volume) containing 1.0 μL of DNA template, 1.0 μL each of forward and reverse primers (10 μM), and 22 μL of 1.1 × T3 Super PCR Mix (Tsingke, Beijing, China) for denaturation. The thermal cycling profile was as follows: 98°C for 2 min; 35 cycles at 98°C for 10 s, 55°C for 10 s, and 72°C for 10 s; and 72°C for 2 min. To identify contamination during PCR, 1 μL of ddH2O was used as the template for each blank. PCR was performed separately for shore, nearshore, and midline samples, with one blank per run, totaling 12 PCR blanks across four sampling events. The PCR products were examined using 2% agarose gel electrophoresis and purified using an AxyPrep DNA Gel Extraction Kit (Axygen, Hangzhou, China). Only samples with a clear target amplicon proceeded to library preparation, sequencing, and downstream analyses. Indexing PCR and library preparation were performed using the NEBNext Ultra DNA Library Prep Kit (New England Biolabs, Ipswich, MA, USA). The libraries were sequenced using a NovaSeq 6000 system for paired‐end 2 × 150 bp reads (Illumina, San Diego, CA, USA). The expected number of total reads per sample was set to 500,000 to reduce the sequencing depth bias effects for each sample.

### 2.3 Bioinformatic Analyses

The paired‐end reads were trimmed and merged using Trimmomatic v.0.36 (Bolger et al. 2014) and Flash (Magoč and Salzberg 2011), with primer‐template mismatches ≤ 2 bp, Phred quality scores ≥ 20, and base overlaps ≥ 10. Merged reads containing ambiguous bases or bases shorter than 100 bp were excluded. Sequences were clustered into operational taxonomic units (OTUs) with 97% identity using Usearch v.10 (Edgar 2010). OTUs with a total read count < 10 among all data were removed, whereas OTUs with 90% coverage, ≥ 97% identity, and ≤ 10^−5^ E‐values were assigned to taxonomic groups using Blastn (Camacho et al. 2009). The reference database constructed by Zhang, Zhao, and Yao (2020), which was created using Actinopterygii 12S rRNA fragments obtained from the EMBL database using Tele02 primers, was used in this study. This database includes 4473 fish species, 33 of which are historically recorded species from Erhai Lake. As of May 2020, complete mitochondrial genome sequences had been published for 34 of the 45 fish species historically recorded in Erhai Lake (Table S1) (Du and Li 2001; Fei et al. 2011; He et al. 2010). Notably, *Schizothorax griseus* was absent from the Zhang, Zhao, and Yao (2020) reference database. To ensure comprehensive coverage, we added the corresponding 12S rRNA sequence for this species to our study.

The annotation results required further manual verification. First, unmatched sequences were excluded. Second, sequences unlikely to be found in Erhai Lake (including marine and freshwater fishes from other regions) were eliminated. For sequences where two or more fishes were identified simultaneously, the higher taxa (e.g., genus, subfamily, and family) to which those species belonged were subsequently determined. Finally, annotated sequences in which the proportion of read counts in a real sample was ≤ 0.1% were filtered out to avoid sequencing errors or contamination (Schnell et al. 2015).

To eliminate potential statistical bias in subsequent analyses, species reads in each eDNA sample were standardized due to variations in sequencing depth across different samples. For standardization, three data forms were employed for downstream statistical analysis: (1) relative read abundance, which represents the ratio of read counts per taxon to the total read counts in a given sample; (2) presence/absence, which is reflected by binary (1–0) data reflecting taxon presence or absence in a sample; and (3) site occupancy, which is defined as the proportion of sampling sites in which a particular taxon is detected out of all sampling sites. The average relative read abundance was calculated for the repeated samples. A taxon was considered present at a specific sampling point if it appeared in any repeated samples.

### 2.4 Statistical Analyses

#### 2.4.1 Community Composition Analysis

A Venn diagram was plotted using the “venn” package in R v.4.1.0 software (http://www.r‐project.org/) to show the number of taxa shared among the four sampling events. Bar histograms were plotted in R using the “*barplot”* function based on the relative read abundance of detected taxa across the four sampling events to illustrate the taxonomic composition and identify dominant taxa.

#### 2.4.2 Rank Abundance Estimation

A Venn diagram was plotted using the “venn” R package to show the overlapping taxa between eDNA monitoring and species records from traditional survey methods. Overlapping taxa were ranked according to three relative abundances—dominant, common, and occasional—based on reports from these traditional surveys (Table S1). To assess whether the eDNA data reflected the rank abundance, the relationship between eDNA data (site occupancy and relative read abundance) and rank abundance from traditional surveys was investigated by calculating Spearman's rank correlations. Data were plotted by fitting a smoothed linear model with the “*geom_smooth”* function (model = lm) in the “ggplot2” R package (Wickham 2011).

#### 2.4.3 eDNA Spatial and Temporal Difference Visualization

This study detected fish during different seasons (i.e., summer and autumn) and at different sampling sites (i.e., shore, nearshore, and midline). To qualitatively and quantitatively visualize the spatial and temporal differences in fish eDNA, this study used ArcGIS v.10.8 (ESRI, Redlands, CA, USA) to create interpolation maps, which revealed differences in taxa numbers across sampling sites between summer and autumn. The “pheatmap” R package (Kolde 2012) was used to create heatmaps showing differences in the relative read abundance across sampling sites between summer and autumn.

#### 2.4.4 α‐Diversity Analysis

The α‐diversity of eDNA data was analyzed using the richness, Shannon, and Simpson indices:

Richness index:

R=S

Shannon index:

$$H^{\prime }=-\sum_{i=1}^{s}P_{i}\ln P_{i}$$

Simpson index:

$$\mathrm{GS}=1-\sum_{i=1}^{S}P_{i}^{2}$$

where *S* is the number of taxa and *P*_*i*_ is the relative read abundance of taxon *i* in a sample. All α‐diversity indices were calculated using the “vegan” R package (Oksanen et al. 2007).

The Kruskal–Wallis test was used to assess significant diversity variations among the four sampling events, two sampling seasons, and three sampling sites, as it is a non‐parametric method suitable for comparing more than two independent groups when the data does not follow a normal distribution. When differences between groups were significant, pairwise comparisons were conducted using the Wilcoxon test. The threshold for significance was *p* < 0.05.

Violin and box plots illustrating group disparities were generated using the “ggpubr” R package (Kassambara 2020). Box plots highlight the median, interquartile range, and outliers, while violin plots display the full data distribution, including density and spread. Together, they provide a more comprehensive visualization of the data's variation, revealing patterns or differences that may not be apparent from a single plot.

#### 2.4.5 β‐Diversity Analysis

Principal coordinate analysis (PCoA) was conducted to elucidate spatiotemporal variations in fish community structure based on a Jaccard distance matrix calculated from taxon presence/absence and a Bray–Curtis distance matrix calculated from taxon relative read abundance. Permutational multivariate analysis of variance (PERMANOVA) was applied to test for differences among groups, and the explained variance (*R* ^2^) and significance were reported (*p* < 0.05). PCoA ordinations were visualized using the “*cmdscale*” R function. The Jaccard and Bray–Curtis dissimilarities and PERMANOVA analysis were performed using the “vegan” R package.

RDA was performed to examine the correlation between environmental factors and fish communities based on eDNA data across seasons and sites. To eliminate the effects of occasional species, seven taxa with a low relative read abundance (< 0.1) were excluded, with 29 taxa retained for analysis. Prior to RDA, all environmental variables were log10 transformed to eliminate dimensional effects (Table S2). In addition, we evaluated collinearity among the 11 environmental variables by examining pairwise Pearson correlation coefficients and calculating variance inflation factors (VIFs). Variables showing strong pairwise collinearity (e.g., *r* ≥ 0.7) and/or high VIF values (e.g., VIF ≥ 5) would be considered for removal or consolidation. In our dataset, collinearity was low (maximum *r* = 0.59; maximum VIF = 2.06, Table S3); therefore, all 11 variables were retained for subsequent RDA. The RDA was performed using the “vegan” R package.

## 3 Results

### 3.1 Sequencing Data and Controls

Across four sampling campaigns (2020–2021), we collected 528 water samples. Of these, 355 were amplified and sequenced successfully and thus were included in the analyses (summer 2020: *n* = 132; autumn 2020: *n* = 52; summer 2021: *n* = 131; autumn 2021: *n* = 40). After Illumina high‐throughput sequencing, ~28.87 million raw reads were obtained for samples from summer 2020, 31.32 million were obtained for autumn 2020, 87.47 million were obtained for summer 2021, and 34.62 million were obtained for autumn 2021. After quality control and filtering, ~25.91 million (summer 2020), 14.22 million (autumn 2020), 48.30 million (summer 2021), and 19.03 million (autumn 2021) clean reads were obtained. After BLAST analysis and manual verification, the proportion of high‐quality sequences identified as fish was approximately 97.30% and 94.89% for summer and autumn 2020, and 97.97% and 86.95% for summer and autumn 2021 (Table S4).

Although some contamination reads were detected in the negative controls of the 2021 summer (samples M2, M12, N3, and N12) and autumn (samples N1, N2, N4, N5, and S4) samples, the number of these reads was consistently much lower than that in the actual samples (2–40 times higher; Table S5), indicating that the contamination was negligible. Only the filtration negative control from sample N6 (autumn 2021) exhibited a two‐fold increase in total read count (141 thousand reads) compared to the corresponding sample. To enhance the robustness of the eDNA data analysis, data from sample N6 (autumn 2021) were excluded. The 355 replicate samples collected from the four sampling events were consolidated into 134 statistical samples and transformed into three standardized data types for each taxon: relative read abundance, presence/absence, and site occurrence.

### 3.2 Fish Taxa Detection With eDNA

Across the four sampling events, 36 fish taxa (27 species‐level taxa +9 higher‐rank taxa) were detected. Because of limitations of the reference database and high sequence similarity among closely related species, nine detections could only be assigned at higher taxonomic ranks, namely the genera *Homatula*, *Siniperca*, *Misgurnus*, *Hypophthalmichthys*, *Rhodeus*, *Schizothorax*, *Rhinogobius*, and *Silurus*, and the subfamily Cyprininae. Overall, the detected taxa spanned 8 orders, 14 families, 26 genera, and 27 species, and comprised five endemic taxa and 31 non‐native taxa (Table 1 and Table S6).

**TABLE 1:** Fish taxa detected using eDNA metabarcoding in four sampling events in Erhai Lake.

Order	Family	Fish taxa	Code	Occurrence			
				Summer		Autumn	
				Jun 2020	Jun 2021	Nov 2020	Sep 2021
				Total sites (44)	Total sites (44)	Total sites (44)	Total sites (44)
Anabantiformes	Channidae	*Channa argus*	CAR	10	12	10	14
Beloniformes	Adrianichthyidae	*Oryzias latipes*	OLA	0	1	0	0
Centrarchiformes	Centrarchidae	*Micropterus salmoides*	MSA	7	12	0	0
	Sinipercidae	*Siniperca* spp.	SIN	1	26	6	0
Cypriniformes	Cobitidae	*Homatula* spp.^a^	HOM	0	8	0	3
		*Misgurnus* spp.	MAN_MDA	23	10	9	6
		*Misgurnus anguillicaudatus* ^a^	MAN	9	3	2	0
		*Misgurnus dabryanus*	MDA	22	9	3	1
	Cyprinidae	*Acheilognathus chankaensis*	ACH	0	0	4	0
		*Carassius auratus* ^a^	CAU	44	44	25	21
		*Ctenopharyngodon idella*	CID	4	19	13	5
		Cyprininae	CAU_CCA	23	28	21	10
		*Cyprinus carpio*	CCA	42	43	22	21
		*Hemiculter leucisculus*	HLE	32	42	20	21
		*Hypophthalmichthys* spp.	HMO_HNO	27	26	8	13
		*Hypophthalmichthys molitrix*	HMO	26	22	8	5
		*Hypophthalmichthys nobilis*	HNO	2	2	1	2
		*Megalobrama amblycephala*	MAM	0	5	6	0
		*Mylopharyngodon piceus*	MPI	0	1	0	0
		*Pseudorasbora parva*	PPA	35	34	16	21
		*Rhodeus* spp.	ROC_RSI	0	2	0	0
		*Rhodeus ocellatus*	ROC	0	2	0	0
		*Rhodeus sinensis*	RSI	27	26	11	21
		*Schizothorax* spp.^a^	SLI_STA	0	4	0	11
		*Schizothorax taliensis* ^b^	STA	0	22	0	0
		*Squaliobarbus curriculus*	SCU	2	0	10	0
Cyprinodontiformes	Poeciliidae	*Gambusia affinis*	GAF	10	6	5	0
Gobiiformes	Odontobutidae	*Micropercops swinhonis*	MSW	29	22	15	21
	Gobiidae	*Rhinogobius* spp.	RCL_RGI	11	10	3	8
		*Rhinogobius cliffordpopei*	RCL	24	30	16	21
		*Rhinogobius giurinus*	RGI	39	35	21	21
Osmeriformes	Osmeridae	*Hypomesus olidus*	HOL	33	35	22	21
	Salangidae	*Neosalanx taihuensis*	NTA	22	21	15	10
Siluriformes	Clariidae	*Clarias fuscus*	CFU	0	0	0	1
	Siluridae	*Silurus* spp.	SIL	9	14	8	21
	Bagridae	*Tachysurus fulvidraco*	TFU	24	33	17	16
		Number of fish taxa	36	26	33	27	24

*Note:* The “Code” refers to the abbreviated Latin names assigned to taxa to simplify the presentation of subsequent analytical results. The fish taxa detected in shore, nearshore, and midline sites of the Erhai Lake in four sampling events are shown in Table S6.

^a^ Native non‐endemic taxa of the Erhai Lake.

^b^ Endemic taxa of the Erhai Lake.

**FIGURE 2:**
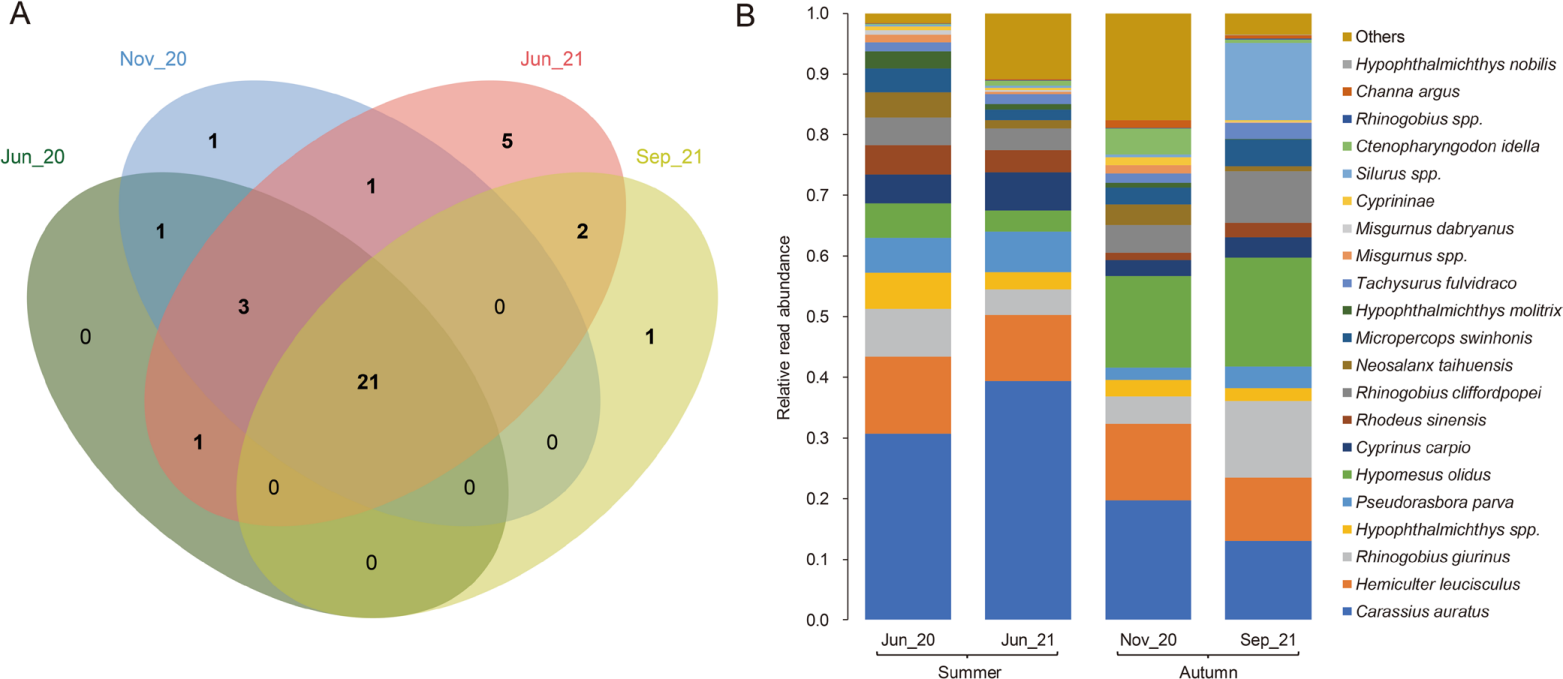
Number (A) and composition (B) of fish taxa detected in four sampling events using eDNA metabarcoding.

A total of 21 taxa were consistently detected across all four summer–autumn sampling events (Figure 2A). Among them, *Carassius auratus* , *Hemiculter leucisculus* , *Rhinogobius cliffordpopei* , *Rhinogobius giurinus* , *Hypomesus olidus* , and *Cyprinus carpio* showed relatively high read abundances and were therefore considered dominant taxa (Figure 2B).

### 3.3 Correlations Between eDNA Data and Long‐Term Data From Traditional Surveys

For comparison with the historical records, we applied a *non‐redundant counting rule* to avoid double‐counting taxa represented at multiple taxonomic ranks in the eDNA dataset. Specifically, among the *nine higher‐rank detections*, *six* (Cyprininae, *Hypophthalmichthys* spp., *Misgurnus* spp., *Rhinogobius* spp., *Rhodeus* spp., and *Schizothorax* spp.) were excluded because corresponding species‐level taxa were also detected, and including both ranks would result in double counting. In contrast, the remaining *three higher‐rank detections* (*Homatula* spp., *Siniperca* spp., and *Silurus* spp.) were *retained* because no species‐level assignments within these genera were recovered by eDNA.

Accordingly, Figure S1 summarizes the non‐redundant taxa used *for the comparison*, including *25 historically recorded taxa* and *five additional taxa detected by eDNA*. To ensure taxonomic consistency between eDNA and historical datasets for downstream analyses, we collapsed the 25 historical taxa into 21 comparable taxa prior to linear fitting and correlation tests. Specifically, *C. auratus* and *C. carpio* were merged as Cyprininae; *H. molitrix* and *H. nobilis* as *Hypophthalmichthys*; *R. cliffordpopei* and *R. giurinus* as *Rhinogobius*; and *S. taliensis* as *Schizothorax*.

**FIGURE 3:**
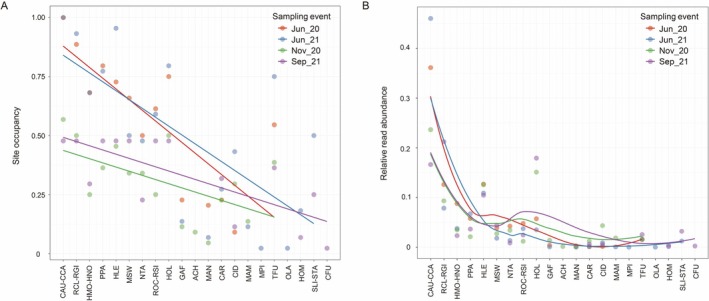
Linear fits of eDNA‐based site occupancy (A) and relative read abundance (B) against rank abundance from traditional monitoring. Fish taxa corresponding to the codes in abscissa are shown in Table 1, in which 21 taxa are ordered according to their long‐term rank abundance reported by traditional monitoring.

Linear fitting showed consistent patterns across the four sampling events for both eDNA‐based site occupancy (Figure 3A) and relative read abundance (Figure 3B), and both aligned closely with rank abundance from traditional monitoring. Spearman's rank correlation analyses further indicated significant associations among eDNA‐based site occupancy, eDNA relative read abundance, and traditional rank abundance (*p* < 0.05; Table 2).

**TABLE 2:** Spearman's rank correlations between eDNA‐based site occupancy, eDNA relative read abundance, and rank abundance from traditional monitoring in recent years.

Sampling event	Site occupancy	Relative read abundance
Jun_2020	rho = −0.736, *S* = 789.66, *p* = 0.003	rho = −0.808, *S* = 822.44, *p* = 0.0005
Nov_2020	rho = −0.533, *S* = 1042.4, *p* = 0.034	rho = −0.549, *S* = 1053.4, *p* = 0.028
Jun_2021	rho = −0.697, *S* = 1935.1, *p* = 0.001	rho = −0.790, *S* = 2040.1, *p* = 5.818e^−05^
Sep_2021	rho = −0.622, *S* = 908.22, *p* = 0.013	rho = −0.609, *S* = 901.29, *p* = 0.016

### 3.4 Fish eDNA Spatiotemporal Variation

**FIGURE 4:**
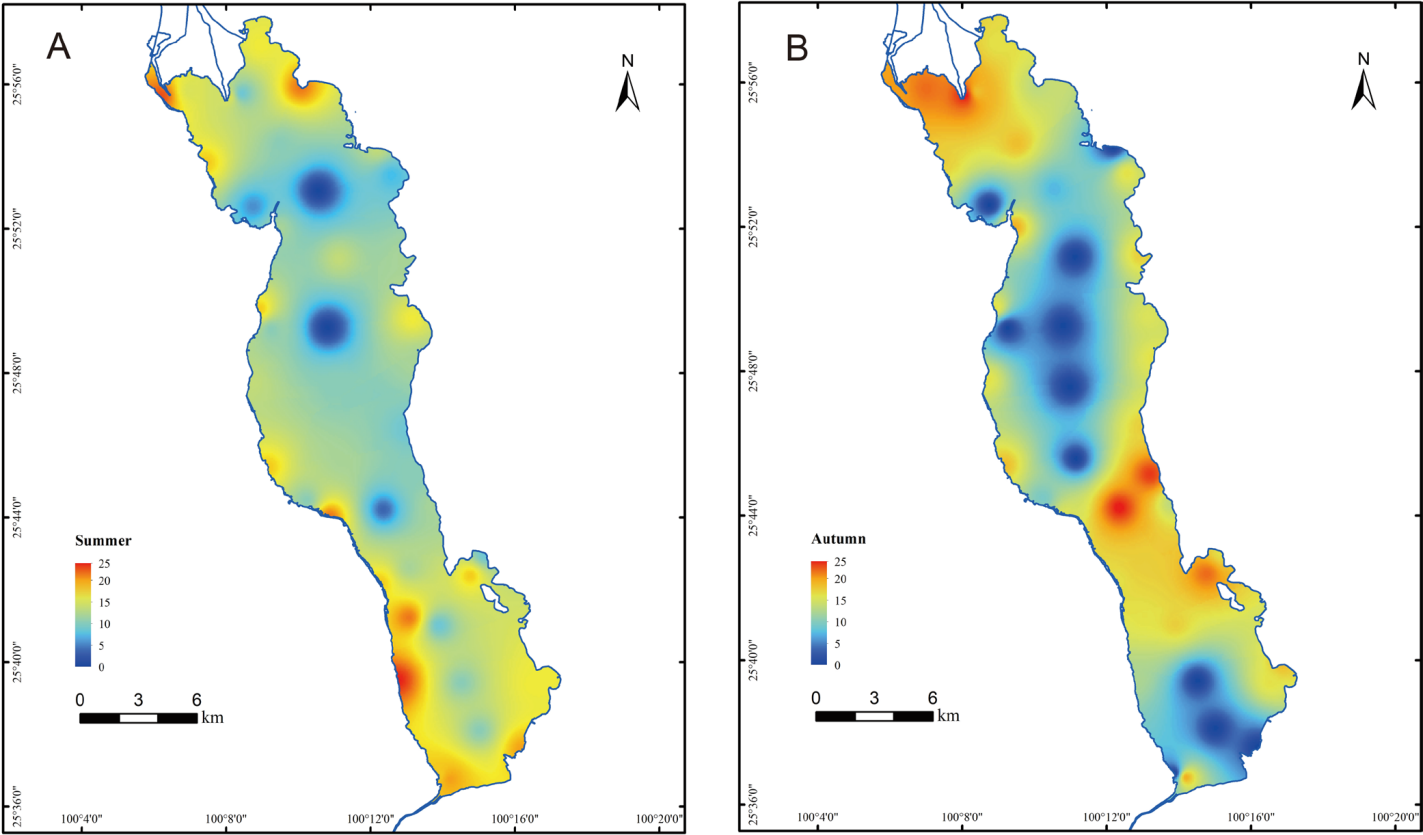
Graphic interpolation of taxa numbers detected at each sampling site during summer (A) and autumn (B). Different colors represent different numbers of detected taxa. Cooler colors indicate fewer taxa, whereas warmer colors indicate more taxa.

**FIGURE 5:**
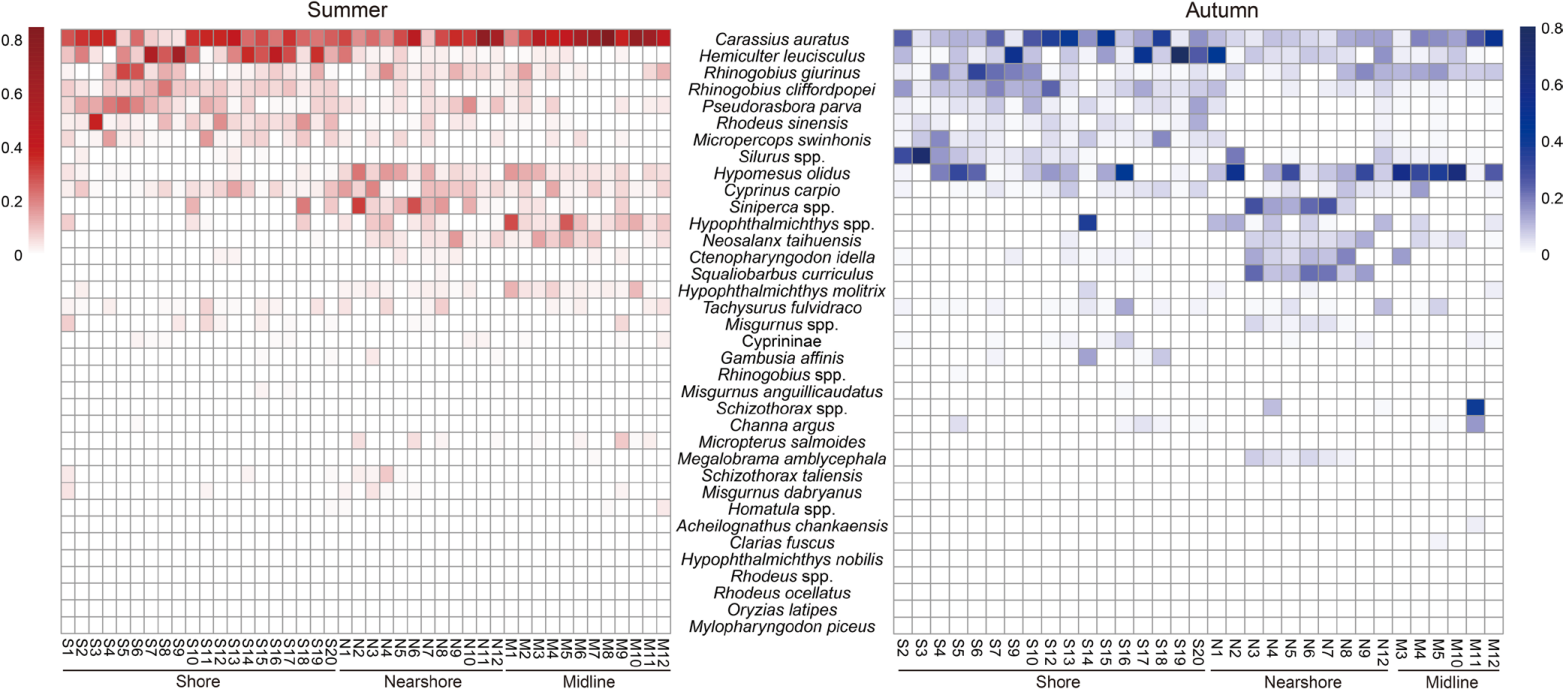
Heatmap of taxa relative read abundance detected at different sampling sites in summer and autumn. Different colors represent different samples across seasons. Abscissa shows samples collected from shore, nearshore, and midline sites. Ordinate shows detected taxa. The deeper color block indicates higher relative read abundance.

Graphical interpolation (Figure 4) and a heatmap (Figure 5) illustrate the spatial and temporal variations in the number and relative read abundance of fish taxa detected using eDNA, respectively. Distinct differences were observed in species detection across seasons and locations (Figure 4). Specifically, more taxa were detected during summer than autumn, and more taxa were found in the shore and nearshore areas than in the midline. Seasonal differences in relative read abundance were evident for certain taxa (Figure 5), such as *C. auratus* , *C. carpio* , *H. leucisculus* , *Pseudorasbora parva* , *Rhodeus sinensis* , *Siniperca* spp., *Hypophthalmichthys* spp., and *Hypophthalmichthys molitrix*, which exhibited higher relative read abundances during summer than during autumn. Conversely, *Silurus* spp., *H. olidus* , *Ctenopharyngodon idella* , and *Squaliobarbus curriculus* showed higher relative read abundances during autumn than during summer. Moreover, distinct variations in relative read abundance between the shore and interior (nearshore and midline) sites were observed for certain taxa, namely, *H. leucisculus* , *R. giurinus* , *R. cliffordpopei* , *P. parva* , *R. sinensis* , *Micropercops swinhonis* , and *Silurus* spp., which displayed higher relative read abundances along the shore than in the interior. *Hypomesus olidus* , *C. carpio* , *Siniperca* spp., *Hypophthalmichthys* spp., *Neosalanx taihuensis* , *C. idella* , *S. curriculus* , and *H. molitrix* had greater relative read abundances from the interior sites than the shore sites.

### 3.5 α‐Diversity

**FIGURE 6:**
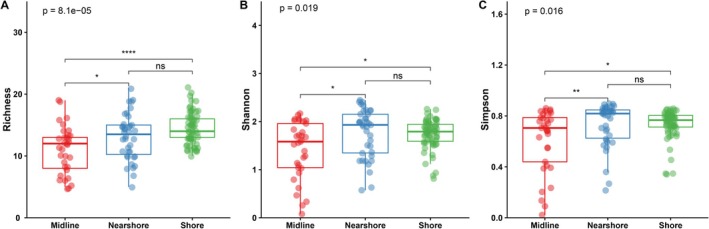
Spatial differences in richness (A), Shannon (B), and Simpson (C) indices. A Kruskal–Wallis test was used to determine whether sampling spaces significantly impact indices (*p* < 0.05 indicates a significant effect of sampling site on the index). The difference between groups was tested using a Wilcoxon test. “ns” represents no significant difference between groups; “*” represents a significant difference between groups (0.01 < *p ≤ 0.05, 0.001 < **p ≤ 0.01, ****p ≤ 0.0001).

The α‐diversity indices of the 134 eDNA samples are presented in Table S7. Only the richness index exhibited significant variations across the four sampling events (*p* < 0.05; Figure S2). Specifically, significant differences were observed between the two summer and autumn sampling events in 2020 and 2021 (*p* < 0.05). When analyzing all eDNA samples from the four sampling events collectively, no significant differences in α‐diversity were observed between summer and autumn (*p* = 0.17 for the richness index, *p* = 0.3 for the Shannon index, *p* = 0.44 for the Simpson index; Figure S3); however, significant variations were found among different sampling sites (*p* < 0.001 for the richness index, *p* < 0.05 for the Shannon index, *p* < 0.05 for the Simpson index; Figure 6). Notably, significantly differences in α‐diversity were not observed between the shore and nearshore areas; however, significantly higher values were observed in both the shore and nearshore areas than the midline locations (Figure S3).

### 3.6 β‐Diversity

PCoA combined with PERMANOVA revealed significant spatiotemporal variations in fish community structure according to both taxon presence/absence and taxon relative read abundance (Figures S4 and S5). Seasonal effects on community structure were significant for both presence/absence (*R* ^2^ = 0.05, *p* = 0.001) and relative read abundance (*R* ^2^ = 0.06, *p* = 0.001). Likewise, sampling site had significant effects on both presence/absence (*R* ^2^ = 0.11, *p* = 0.001) and relative read abundance (*R* ^2^ = 0.15, *p* = 0.001). Pairwise comparisons among sites showed significant differences between shore and nearshore areas (*R* ^2^ = 0.08, *p*_adj_ = 0.003; *R* ^2^ = 0.11, *p*_adj_ = 0.003), shore and midline areas (*R* ^2^ = 0.11, *p*_adj_ = 0.003; *R* ^2^ = 0.16, *p*_adj_ = 0.003), and nearshore and midline areas (*R* ^2^ = 0.05, *p*_adj_ = 0.003; *R* ^2^ = 0.06, *p*_adj_ = 0.003).

**FIGURE 7:**
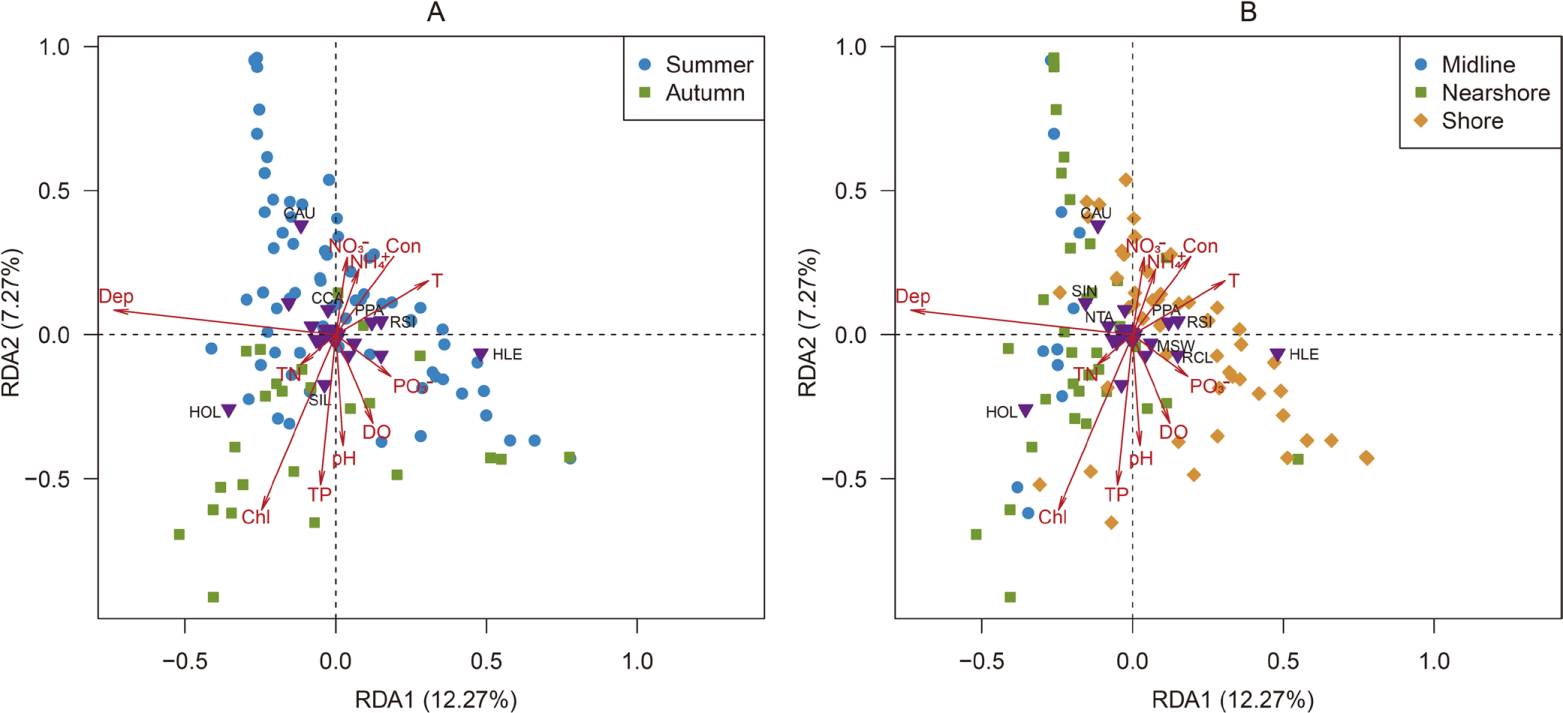
Redundancy analysis (RDA) ordinations of fish communities and environmental factors across different seasons (A) and sites (B). Circles, squares, and diamonds represent samples. Arrows represent environmental factors, including water temperature (T), dissolved oxygen (DO), conductivity (Con), pH, water depth (Dep), nitrate (NO_3_^−^), phosphite (PO_3_^−^), ammonium salt (NH_4_^+^), total nitrogen (TN), total phosphorus (TP), and chlorophyll‐a (Chl). The inverted triangles represent fish taxa. The taxa corresponding to the codes are shown in Table 1.

The RDA revealed significant relationships (*p* < 0.001) between the 26 taxa of fish and 11 environmental factors, which explained 19.54% of the variation in the first two axes (Figure 7). Table 3 presents the correlation coefficients and *p* values, which indicate the impact of environmental factors on the RDA ordination results. Water temperature, conductivity, pH, water depth, total phosphorus, and chlorophyll‐a significantly influenced the ordination patterns. Seasonal distributions of fish communities exhibited strong associations with water temperature, conductivity, pH, total phosphorus, and chlorophyll‐a (Figure 7A). For example, *C. auratus* , *C. carpio* , *H. leucisculus* , *P. parva* , and *R. sinensis* showed higher abundances in summer than in autumn and were closely linked to areas characterized by elevated water temperature and conductivity; and *Silurus* spp. and *H. olidus* were more prevalent in autumn than in summer and associated with areas exhibiting high pH, total phosphorus, and chlorophyll‐a concentrations. Fish community spatial distribution patterns were influenced by water depth (Figure 7B). For example, *Siniperca* spp., *N. taihuensis* , and *H. olidus* were predominantly distributed in deep sections within the interior; *H. leucisculus* , *P. parva* , *R. sinensis* , *R. cliffordpopei* , and *M. swinhonis* were primarily found along the shore (shallower water); and *C. auratus* was distributed along the shore and within the interior. However, the occurrence of *C. auratus* was also influenced by variations in water depth.

**TABLE 3:** Correlation coefficients and *p* values of environmental factors to RDA ordination results.

	RDA1	RDA2	*R* ^2^	*p*
T	0.902	0.431	0.11	0.006**
DO	0.411	−0.911	0.05	0.105
Con	0.779	0.627	0.08	0.026*
pH	−0.177	−0.984	0.07	0.037*
Dep	−0.995	−0.102	0.45	0.001***
NO_3_^−^	0.453	0.891	0.04	0.155
PO_3_^−^	0.947	−0.320	0.03	0.212
NH_4_^+^	0.617	0.787	0.04	0.170
TN	−0.857	−0.516	0.02	0.387
TP	−0.400	−0.916	0.15	0.003**
Chl‐a	−0.658	−0.753	0.28	0.001***

*Note:* Significant codes: 0.01 < *p ≤ 0.05, 0.001 < **p ≤ 0.01, ***p ≤ 0.001.

## 4 Discussion

### 4.1 eDNA Sampling Strategies for Lake Fish

Shoreline sampling is commonly preferred for biodiversity monitoring in aquatic ecosystems due to its safety and cost‐effectiveness (Biggs et al. 2015; Fujii et al. 2019; Valentini et al. 2016). However, while shoreline sampling offers several advantages, it may not fully capture the spatial distribution patterns of fish species, particularly in large lakes. These patterns are influenced by various factors such as habitat preference, interspecific competition, and predation risks. As a result, fish species with patchy distributions may show uneven eDNA presence in lake waters, especially in areas with limited mixing (Ross 2013). This uneven distribution can directly affect the accuracy of fish diversity detection using eDNA methods. Moreover, shoreline sampling alone may not be sufficient to detect fish species inhabiting deeper waters, which are often underrepresented in such samples (Hänfling et al. 2016; Handley et al. 2019; Zhang, Lu, et al. 2020). Therefore, to achieve more comprehensive monitoring, alternative sampling strategies may be necessary, especially when specific monitoring objectives are involved (Hänfling et al. 2016; Sato et al. 2017).

Significant differences were observed in three α‐diversity indices between sampling sites, indicating variations in detected fish diversity across areas. This study revealed regionalized distribution patterns of fish eDNA within a large lake and emphasized that fine‐scale spatial sampling is necessary to capture fish eDNA from diverse habitats within still water bodies. This comprehensive sampling approach ensured reproducible results when eDNA was detected from multiple replicates. Additionally, spatial variation in α‐diversity revealed that fish diversity observed along the shoreline and nearshore areas was essentially equivalent and significantly higher than that observed in the midline area. These findings highlight the efficacy of shoreline sampling as a cost‐effective strategy for the detection of various fish species within lakes. The shoreline sampling approach was particularly effective in capturing eDNA from most fish species, probably because of the abundant aquatic vegetation present in these shallow waters (Zhou et al. 2016). This study is consistent with that of Handley et al. (2019) and Zhang, Lu, et al. (2020), who validated the feasibility of shoreline sampling for assessing fish diversity in large lakes and the need for fine‐scale spatial sampling to reveal the spatial distribution of fish in lakes.

### 4.2 Fish Diversity

The eDNA analysis conducted in this study revealed the presence of 36 fish taxa, with the Cyprinidae family emerging as the dominant group, accounting for 50% of all identified taxa. Notably, non‐native taxa comprised a significant proportion (86.11%) of the total fish taxa, which is consistent with findings from traditional fish monitoring studies (Yan et al. 2012; Zhou et al. 2016; Zhou 2018; Tang et al. 2013). By utilizing repeated seasonal and fine‐scale spatial sampling, this study provided a more comprehensive assessment of fish diversity compared to Shu et al. (2020), who identified 17 fish taxa across 16 sampling sites, including 12 shoreline sites and 4 midline sites. In total, we identified 25 historically recorded fish taxa, although 20 additional taxa, also historically recorded, were not detected. Among the undetected taxa, 10 species lacked available reference sequences, preventing the accurate annotation of eDNA sequences even when these species were present in the samples. The remaining 10 species had reference sequences; however, most were endangered native species with significantly lower abundance compared to non‐native species, making their detection more challenging.

In this study, certain closely related species, such as *Misgurnus anguillicaudatus* and *Misgurnus dabryanus*, could not be differentiated when the Tele02 primers were used to amplify the 12S rRNA marker. Low species resolution due to short amplified fragments is a known challenge in eDNA metabarcoding (Hänfling et al. 2016; Valentini et al. 2016; Yamamoto et al. 2017; Zhang, Lu, et al. 2020). While these challenges highlight certain limitations of the method, they also emphasize the need for ongoing improvements in technical aspects, such as primer design, sampling strategies, and reference databases. Despite these challenges, eDNA metabarcoding remains a highly effective and non‐invasive tool for monitoring fish diversity, especially in large and complex ecosystems like Erhai Lake. Future advancements in primer specificity and reference database expansion will further enhance species detection and overall accuracy.

Although eDNA metabarcoding does not accurately reflect the true abundance or biomass of a species based on read abundance, several studies have demonstrated significant correlations between site occupancy or relative read abundance detected using eDNA and the long‐term rank abundance of species detected using traditional monitoring methods (Hänfling et al. 2016; Handley et al. 2019; Thomsen et al. 2016). Similarly, this study revealed significant correlations between site occupancy and relative read abundance based on eDNA detection and the fish rank abundance reported from traditional monitoring approaches. These findings highlight the potential of eDNA metabarcoding for assessing fish rank abundance within aquatic ecosystems.

### 4.3 Temporal and Spatial Distribution of Fish

The β‐diversity analysis revealed significant spatial and temporal variations in the structure of fish communities. The seasonal distribution of fish communities was primarily influenced by water temperature and nutrient status (total phosphorus and chlorophyll‐a). Throughout the year, Erhai Lake maintains a water temperature of 10°C–25°C, with higher temperatures during spring and summer than during autumn and winter. Traditional fish monitoring studies have identified water temperature as a key factor affecting the seasonal distribution patterns of fish communities in Erhai Lake. Feeding intensity, activity level, and reproductive rate tend to increase with rising water temperature during spring and summer; therefore, most fish species exhibit higher densities and biomass during the seasons when they are more susceptible to capture (Tang et al. 2013; Zhou et al. 2016). These findings are consistent with the eDNA results, which identified a greater number of fish taxa in summer than in autumn. Moreover, the RDA revealed that certain Cyprinidae species (i.e., *C. auratus* , *C. carpio* , *H. leucisculus* , *P. parva* , and *R. sinensis* ) were more widely distributed in warmer months when the water temperature was higher. Total phosphorus and chlorophyll‐a in the water column serve as crucial indicators for assessing the trophic status of lake waters. The environmental factor data obtained in this study suggested that water bodies underwent greater eutrophication in autumn than in summer. Traditional fish monitoring studies have demonstrated that variations in fish community distribution in Erhai Lake are influenced by changes in the trophic conditions of the water body (Zhou et al. 2016). When the nutrient status of a water body is elevated, phytoplankton growth is accelerated, leading to an increase in populations of small phytoplankton‐feeding fish. Species with high pollution tolerance and ecological niche competition thrive under these conditions. Thus, the findings obtained in this study are consistent with the distribution pattern observed by Zhou et al. (2016). The eDNA data in this study revealed that *Silurus* spp. (a taxon with high pollution tolerance) and *H. olidus* (a species that feeds on plankton) exhibited a higher read abundance during autumn than summer. Additionally, the RDA revealed a broader spatial distribution of the two taxa during periods characterized by elevated trophic states in the water column.

Water depth was the main factor that influenced the spatial distribution of fish communities in this study. Consistent with these findings, fish monitoring studies have demonstrated that water depth significantly affects the spatial distribution of fish in Erhai Lake (Fei et al. 2012; Zhou et al. 2016). The shallow waters of Erhai Lake are abundant in aquatic vegetation, shoals, and bays, which provide hiding places and feeding grounds for most small‐ and medium‐sized fishes, such as *C. auratus* , *H. leucisculus* , *P. parva* , *R. cliffordpopei* , and *R. giurinus* . Conversely, larger species such as *C. carpio* , *H. molitrix* , *H. nobilis* , and *C. idella* are predominantly found in open water, where they experience minimal disturbance from human activities.

*Neosalanx taihuensis* and *H. olidus* are small fish species that primarily feed on zooplankton in open water (Zhou et al. 2016). Previous studies (Fei et al. 2012; Zhou et al. 2016) have shown that zooplankton density is higher in the open water of Erhai Lake compared to the shoreline, which likely explains the dominant occurrence of these two species in open water. The eDNA data also revealed that fish inhabiting shallow water exhibited higher relative read abundance in the shore samples while those residing in open water displayed greater relative read abundance in the interior samples. The RDA further confirmed that *H. leucisculus* , *P. parva* , *R. sinensis* , *R. cliffordpopei* , and *M. swinhonis* were predominantly distributed along the shore while *Siniperca* spp., *N. taihuensis* , and *H. olidus* were predominantly distributed in the interior.

Overall, the fish spatiotemporal distribution based on differences in eDNA across seasons and sites was generally consistent with that observed using traditional fish monitoring methods. Thus, eDNA techniques can effectively monitor the spatiotemporal distribution of fish in large lakes. The mechanisms underlying spatiotemporal heterogeneity of fish eDNA in aquatic ecosystems are complex (Lacoursiere‐Roussel and Deiner 2021). For example, studies have demonstrated that hydrological features and fish ecological characteristics can markedly influence the spatiotemporal distribution of fish eDNA (Bracken et al. 2019; Deiner and Altermatt 2014; Stoeckle et al. 2017). Additionally, other dynamic factors such as water temperature, pH, and trophic status may play a role in influencing the release and degradation of fish eDNA (Barnes et al. 2014; Eichmiller et al. 2016). Therefore, developing a comprehensive understanding of how lake hydrological characteristics and different habitats impact the spatiotemporal distribution of fish eDNA will optimize sampling strategies and enhance interpretation accuracy when analyzing eDNA data. Further investigations are required to explore these aspects.

### 4.4 Suggestions for Monitoring Rare Fish in Erhai Lake

Erhai Lake contains 45 recorded fish species, including 17 native species, among which 7 are classified as endangered. Most endemic species have not been captured for many years, including *S. taliensis*, *Barbodes daliensis*, and *Poropuntius exiguous* (Yan et al. 2012); therefore, these species represent key conservation targets for maintaining fish diversity. Invasive species are a major cause of the decline in native fish diversity in Erhai Lake (Du and Li 2001). However, traditional monitoring methods often fail to detect non‐native species during the early stages of invasion, particularly when they occur at low abundance. Improving detection sensitivity for low‐abundance non‐native species and developing an early‐warning system remain important challenges for conservation management. Many studies have consistently demonstrated that eDNA technology is superior to traditional methods for the detection of rare species. The heightened detection sensitivity of eDNA‐based methods enables effective monitoring of low‐abundance species that are difficult to capture using conventional approaches (Keskin et al. 2016; Evans et al. 2017; Balasingham et al. 2018). In this study, eDNA technology successfully detected one endangered endemic fish (*S. taliensis*) and five previously unreported non‐native fish taxa (*M. dabryanus*, *Micropterus salmoides* , *S. curriculus* , *Silurus* spp., and *Siniperca* spp.). These findings underscore the immense potential of eDNA metabarcoding for monitoring both endangered and non‐native species in large lake ecosystems.

However, for eDNA technology to reach its full potential, the establishment of comprehensive reference sequence database is crucial. Accurate species identification depends on well‐curated reference sequences, and expanding these databases for native and non‐native species in Erhai Lake is essential for reliable detection. The success of eDNA in identifying rare and invasive species underscores its potential in early warning systems, enabling timely management actions to prevent biodiversity loss. By combining eDNA monitoring with traditional methods, ecological professionals can track water bodies with detected endangered or non‐native species and implement targeted conservation measures.

## Author Contributions

**Lu Shu:** formal analysis (lead), investigation (lead), writing – original draft (lead). **Arne Ludwig:** investigation (supporting), methodology (supporting), writing – review and editing (equal). **Hongmei Pan:** investigation (equal). **Jiayan Lin:** investigation (equal). **Yuan Xu:** investigation (equal). **Hang Shan:** investigation (equal). **Te Cao:** conceptualization (equal), resources (lead), writing – review and editing (equal). **Zuogang Peng:** conceptualization (lead), funding acquisition (lead), project administration (lead), writing – review and editing (lead).

## Funding

This work was supported by the grant from the National Key Research and Development Program of China (No. 2018YFD0900805).

## Conflicts of Interest

Zuogang Peng reports financial support was provided by Ministry of Science and Technology of the People's Republic of China. Zuogang Peng reports a relationship with National Natural Science Foundation of China that includes: funding grants. If there are other authors, they declare that they have no known competing financial interests or personal relationships that could have appeared to influence the work reported in this paper.

## Supporting information


**Figure S1:** Venn diagram comparing fish taxa detected in the eDNA survey with those documented in historical records.

**Figure S2:** Differences in Richness (A), Shannon (B), and Simpson (C) indices among the four sampling events. A Kruskal–Wallis test was used to determine whether sampling events had significant impacts on the indices (*p* ≤ 0.05 indicated that sampling events have significant impacts on the index). The difference between groups was tested using the Wilcoxon test. “ns” represents no significant difference between groups; “*” represents a significant difference between groups (0.01 < *p ≤ 0.05, 0.001 < **p ≤ 0.01, ***p ≤ 0.001).

**Figure S3:** Seasonal differences in richness (A), Shannon (B), and Simpson (C) indices. The difference between groups was tested using a Wilcoxon test. Significance level was *p* < 0.05.

**Figure S4:** Principal coordinates analysis (PCoA) ordinations of seasonal differences in fish community structure based on taxon presence/absence (A) and relative read abundance (B). Red and blue points represent summer and autumn samples, respectively. The ellipse indicates the 95% confidence interval. The R^2^ and *p* values were tested using PERMANOVA.

**Figure S5:** Principal coordinates analysis (PCoA) ordinations of spatial differences in fish community structure based on taxon presence/absence (A) and relative read abundance (B). Green, blue, and red points represent shore, nearshore and midline samples, respectively. The ellipse indicates the 95% confidence interval. The *R* ^2^ and *p* values were tested using PERMANOVA.

**Table S1:** ece373082‐sup‐0006‐TableS1.pdf.

## Data Availability

All eDNA metabarcoding sequence data is available on the NCBI Sequence Read Archive (SRA) database (Accession numbers: SRR34080771‐SRR34081135).
