# Supplementary figures and images for: Diversity and Spatiotemporal Distribution of Fish in a Highland Lake in China Based on Environmental DNA Metabarcoding
Source: Ecol Evol. 2026 Feb 11;16(2):e73082. doi: 10.1002/ece3.73082 (PMC12893789; doi:10.1002/ece3.73082)

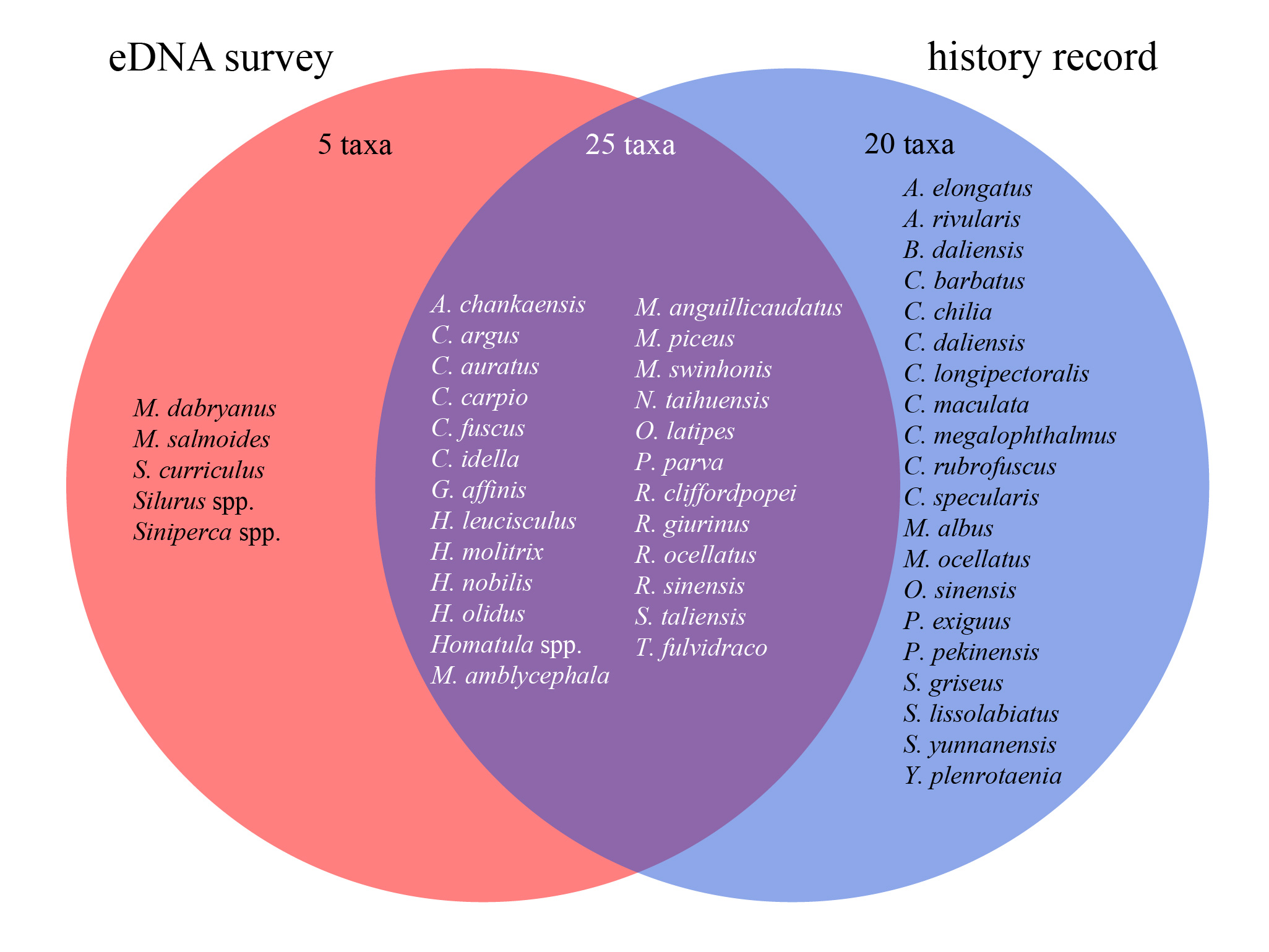

Supplement: Supplementary file 1 — Figure S1: Venn diagram comparing fish taxa detected in the eDNA survey with those documented in historical records. [file ECE3-16-e73082-s005.jpg]

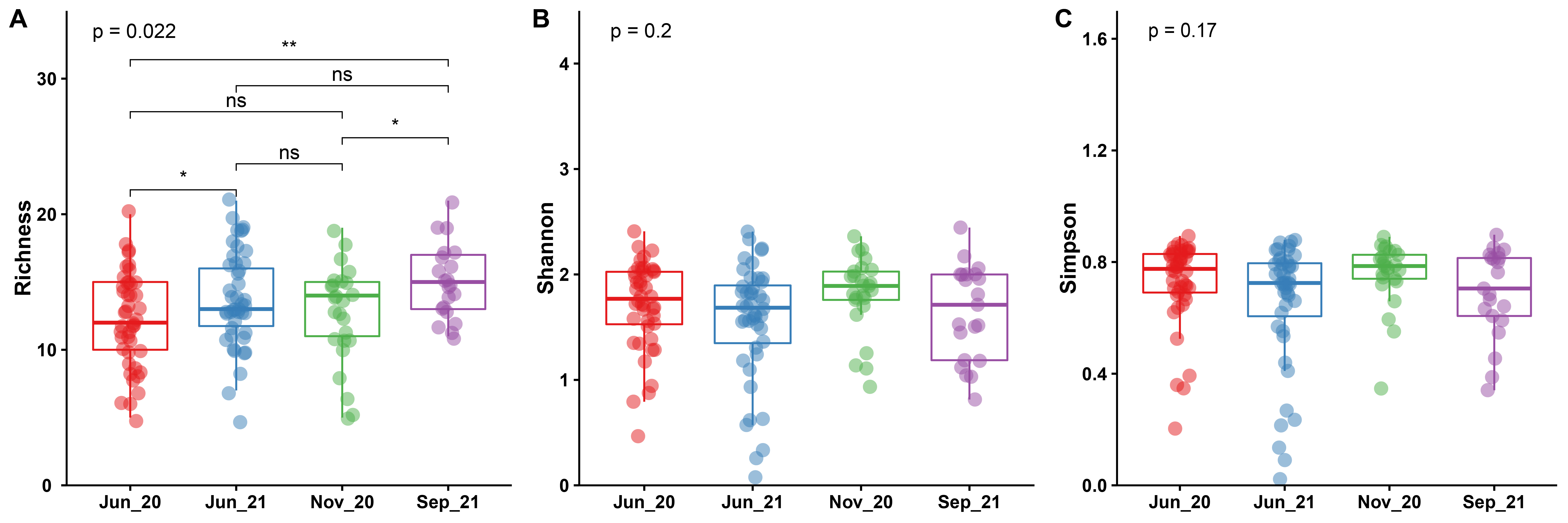

Supplement: Supplementary file 2 — Figure S2: Differences in Richness (A), Shannon (B), and Simpson (C) indices among the four sampling events. A Kruskal–Wallis test was used to determine whether sampling events had significant impacts on the indices (p ≤ 0.05 indicated that sampling events have significant impacts on the index). The difference between groups was tested using the Wilcoxon test. “ns” represents no significant difference between groups; “*” represents a significant difference between groups (0.01 < *p ≤ 0.05, 0.001 < **p ≤ 0.01, ***p ≤ 0.001). [file ECE3-16-e73082-s006.jpg]

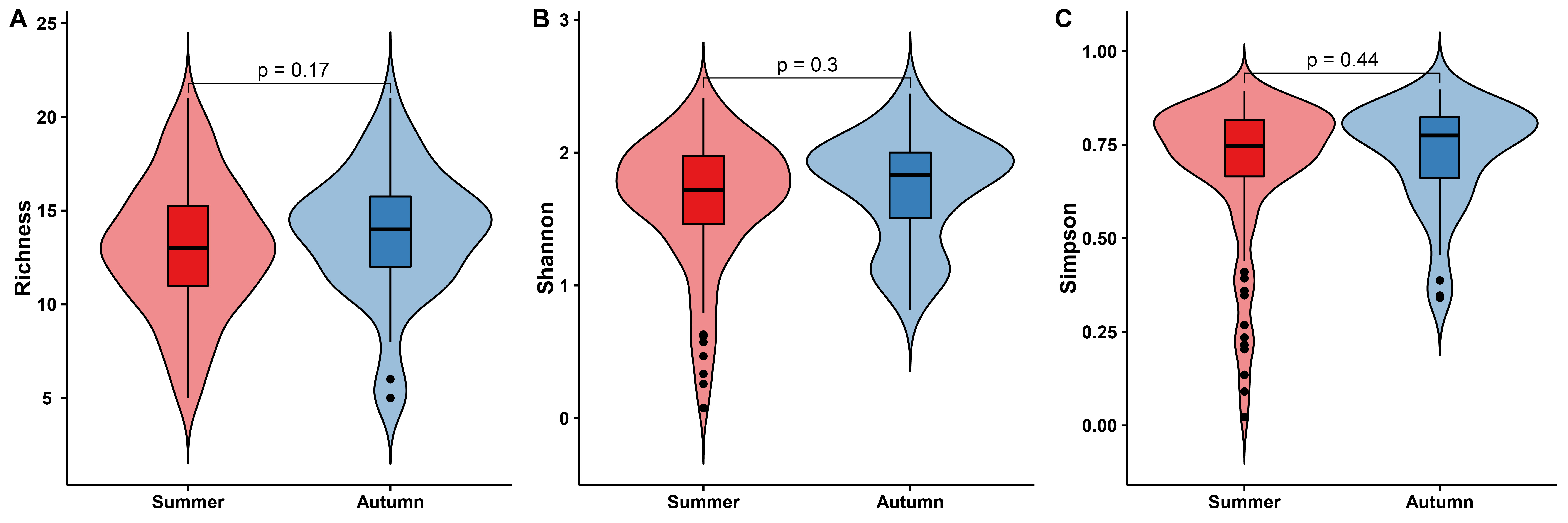

Supplement: Supplementary file 3 — Figure S3: Seasonal differences in richness (A), Shannon (B), and Simpson (C) indices. The difference between groups was tested using a Wilcoxon test. Significance level was p < 0.05. [file ECE3-16-e73082-s002.jpg]

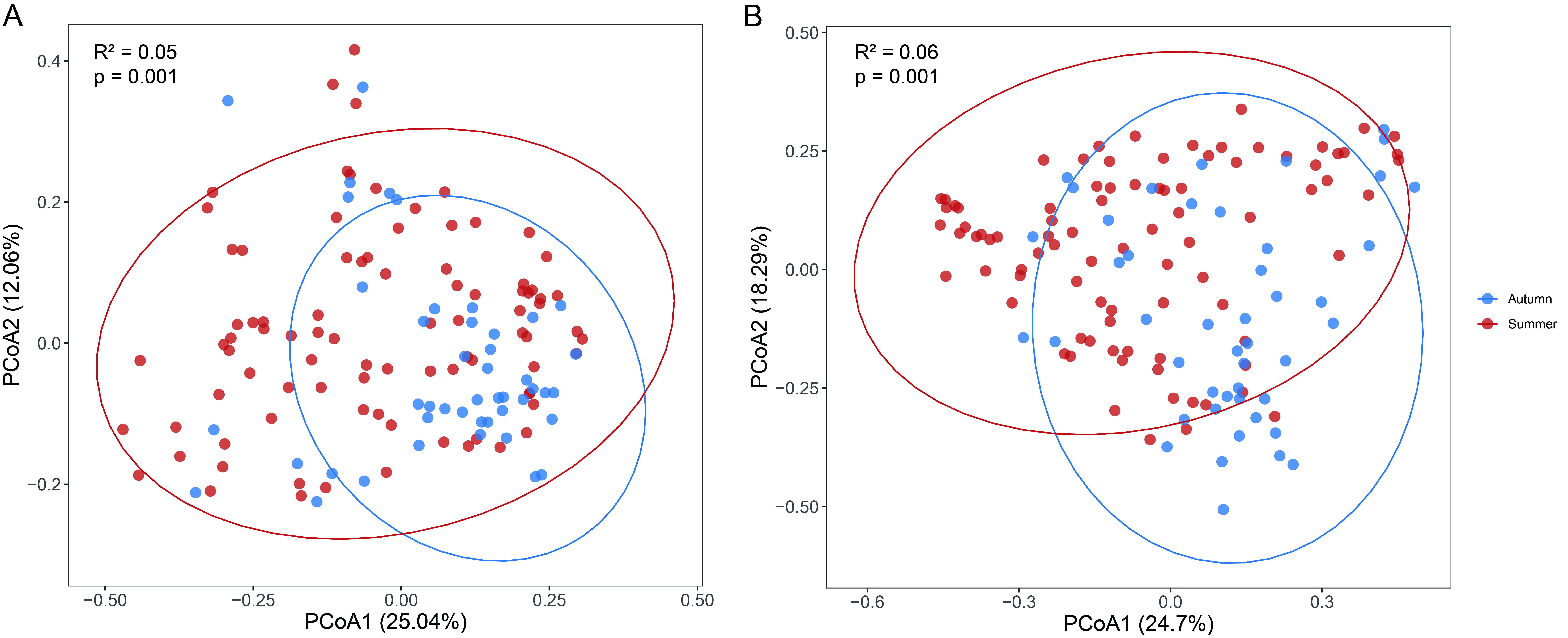

Supplement: Supplementary file 4 — Figure S4: Principal coordinates analysis (PCoA) ordinations of seasonal differences in fish community structure based on taxon presence/absence (A) and relative read abundance (B). Red and blue points represent summer and autumn samples, respectively. The ellipse indicates the 95% confidence interval. The R2 and p values were tested using PERMANOVA. [file ECE3-16-e73082-s003.jpg]

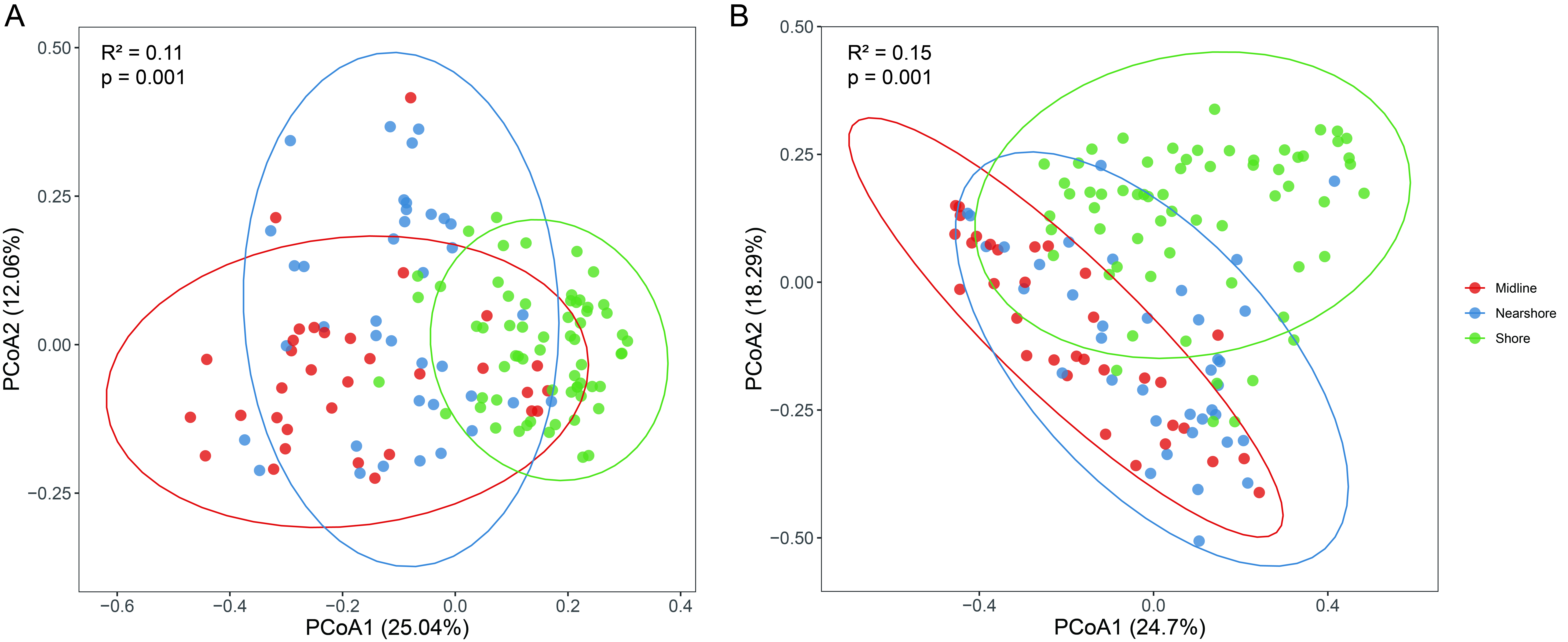

Supplement: Supplementary file 5 — Figure S5: Principal coordinates analysis (PCoA) ordinations of spatial differences in fish community structure based on taxon presence/absence (A) and relative read abundance (B). Green, blue, and red points represent shore, nearshore and midline samples, respectively. The ellipse indicates the 95% confidence interval. The R 2 and p values were tested using PERMANOVA. [file ECE3-16-e73082-s001.jpg]
