# Supplementary material for: Diversity and Spatiotemporal Distribution of Fish in a Highland Lake in China Based on Environmental DNA Metabarcoding
Source: Ecol Evol. 2026 Feb 11;16(2):e73082. doi: 10.1002/ece3.73082 (PMC12893789; doi:10.1002/ece3.73082)
Supplement: Supplementary file 6 — Table S1: ece373082‐sup‐0006‐TableS1.pdf. [file ECE3-16-e73082-s004.pdf]

Table S1. The Fish diversity in the Erhai Lake monitored by traditional fish surveys.

Family	Species [a]	Species rank abundance [b]	GenBank accession No. of mtDNA [c]
Adrianichthyidae	*Oryzias latipes*	occasional	AP004421
Adrianichthyidae	*Oryzias sinensis**	occasional	GU013788
Bagridae	*Tachysurus fulvidraco*	occasional	MH192350
Channidae	*Channa argus*	occasional	KC823605
Channidae	*Channa maculata*	occasional	KC823606
Clariidae	*Clarias fuscus*	occasional	KM029965
Cobitidae	*Misgurnus anguillicaudatus**	common	MK093946
Cobitidae	*Yunnanilus plenrotaenia**	occasional	
Cobitidae	*Homatula anguillioides**	occasional	
Cyprinidae	*Abbottina rivularis*	occasional	KJ183133
Cyprinidae	*Acheilognathus chankaensis*	common	KF695385
Cyprinidae	*Acheilognathus elongatus*	common	
Cyprinidae	*Barbodes daliensis***	occasional (	
Cyprinidae	*Poropuntius exiguous***	occasional (	
Cyprinidae	*Carassius auratus**	dominant	KJ874430
Cyprinidae	*Ctenopharyngodon idella*	occasional	MG827396
Cyprinidae	*Cyprinus carpio*	dominant	MK088487
Cyprinidae	*Cyprinus barbatus***	occasional (	
Cyprinidae	*Cyprinus chilia**	occasional	
Cyprinidae	*Cyprinus daliensis***	occasional (	
Cyprinidae	*Cyprinus longipectoralis***	occasional (	
Cyprinidae	*Cyprinus megalophthalmus***	occasional (	KR869143
Cyprinidae	*Cyprinus rubrofuscus*	occasional	
Cyprinidae	*Cyprinus specularis*	occasional	
Cyprinidae	*Hypophthalmichthys molitrix*	dominant	EU315941
Cyprinidae	*Hypophthalmichthys nobilis*	dominant	HM162839
Cyprinidae	*Megalobrama amblycephala*	occasional	AP011219
Cyprinidae	*Mylopharyngodon piceus*	occasional	AP011216
Cyprinidae	*Pseudorasbora parva*	dominant	JF802126
Cyprinidae	*Rhodeus ocellatus*	common	KT004415
Cyprinidae	*Rhodeus sinensis*	common	KF533721
Cyprinidae	*Schizothorax griseus**	occasional	MF688995
Cyprinidae	*Schizothorax lissolabiatus**	occasional	KF752480
Cyprinidae	*Schizothorax taliensis***	occasional (	MH094667
Cyprinidae	*Schizothorax yunnanensis**	occasional	KP892531
Cyprinidae	*Parabramis pekinensis*	occasional	MK421554
Cyprinidae	*Hemiculter leucisculus*	dominant	KF647872
Gobiidae	*Rhinogobius cliffordpopei*	dominant	KX898434
Gobiidae	*Rhinogobius giurinus*	dominant	KU871066
Odontobutidae	*Micropercops swinhonis*	dominant	KF040334
Osmeridae	*Hypomesus olidus*	common	KP281293
Osphronemidae	*Macropodus ocellatus*	occasional	KJ813282
Poeciliidae	*Gambusia affinis*	common	AP004422
Salangidae	*Neosalanx taihuensis*	dominant	MH348204
Synbranchidae	*Monopterus albus**	occasional	AP002945

a Fish species of the Erhai Lake reported by historical traditional surveys (Du et al., 2001; He et al., 2010; Fei et al., 2011; Yan et al., 2012; Zhou et al., 2016; Zhou et al., 2018; Tang et al., 2013).

b Fish rank abundance monitored by traditional surveys in recent years (Fei et al., 2011; Fei et

For Review Only

b Fish rank abundance monitored by traditional surveys in recent years (Fei et al., 2011; Fei et al., 2012; Yan et al., 2012; Zhou et al., 2016; Tang et al., 2013).
c Blank means no mitochondrial genome sequences published.
* Native species of the Erhai Lake
** Endemic species of the Erhai Lake

For Review Only

Table S2. Log10-transformed environmental variables for each sampling unit used in the redundancy temperature; DO, dissolved oxygen; Con, conductivity; Dep, water depth; NO_3^-, nitrate; PO_3^-, phospha nitrogen; TP, total phosphorus; Chl-a, chlorophyll-a.

sample	T(°C)	DO(mg/L)	Con(μS/cm)	pH	Dep (m)	NO3 (mg/L)	PO3 (mg/L)	NH4 (mg/L)
J20_Ms	-0.50	-0.66	0.75	-0.46	0.30	0.46	-0.01	-0.21
J20_Mm	0.33	0.28	1.17	0.57	2.37	0.46	-0.01	0.43
J20_Mn	0.28	0.43	0.88	0.74	1.07	0.16	1.16	-1.48
J20_N1	0.74	0.76	0.93	0.14	-0.37	0.16	-1.17	-1.85
J20_N2	1.00	-0.36	1.19	0.83	0.43	2.58	-0.01	-0.21
J20_N3	0.33	-1.01	1.17	0.05	0.69	0.46	1.16	-0.84
J20_N4	0.38	-0.01	1.15	0.10	0.96	-0.14	1.16	-0.66
J20_N5	0.33	0.37	1.22	0.23	0.96	0.16	-0.59	-0.48
J20_N6	-0.35	-0.30	0.80	-0.25	1.76	0.46	-0.01	-0.39
J20_N7	-0.35	-0.21	0.81	-0.46	0.69	0.76	-0.59	-0.21
J20_N8	0.28	-0.13	1.16	-0.63	0.43	0.46	1.16	1.70
J20_N9	-0.35	-0.06	0.69	-0.03	0.69	1.07	-0.59	1.52
J20_N10	0.48	0.75	1.03	0.31	2.02	0.76	-0.59	2.25
J20_N11	-0.29	0.67	0.68	0.57	-0.10	0.16	-1.17	-1.66
J20_N12	0.33	-0.09	0.88	0.01	-0.10	0.46	-1.17	-0.84
J20_S1	-0.29	-0.24	0.79	-0.72	-0.77	0.46	1.16	0.52
J20_S2	-0.19	0.19	0.86	-0.03	-0.77	0.76	1.16	-0.57
J20_S3	0.59	0.58	1.13	0.35	-0.77	0.16	-0.59	-0.21
J20_S4	0.22	-0.37	1.09	-0.25	-0.77	0.46	-0.59	0.98
J20_S5	0.33	-0.23	1.14	0.01	-0.77	0.16	-0.59	-0.21
J20_S6	0.12	-0.87	1.06	-0.03	-0.77	-0.14	-0.59	-0.30
J20_S7	1.26	0.01	1.45	-0.33	-0.77	0.46	-0.01	-1.03
J20_S8	1.26	0.07	1.07	1.26	-0.77	2.88	-0.01	-0.12
J20_S9	0.17	0.11	0.87	0.57	-0.77	0.16	-0.01	-0.39
J20_S10	0.28	0.35	0.92	0.57	-0.77	0.46	-0.01	-1.57
J20_S11	-0.45	-0.15	0.76	-0.25	-0.90	0.46	-0.59	-0.21
J20_S12	-0.66	-1.26	0.70	-0.67	-0.90	0.46	-0.59	0.07
J20_S13	0.07	-0.55	1.11	-1.19	-0.90	0.76	4.65	1.61
J20_S14	-0.66	-0.30	0.71	-0.55	-0.90	1.07	-0.59	3.70
J20_S15	-0.19	-0.45	0.56	-0.63	-0.90	5.90	3.49	0.98
J20_S16	-0.29	-0.07	0.78	0.10	-0.90	0.16	-0.59	1.79
J20_S17	0.28	0.65	0.93	0.23	-0.90	0.76	-0.59	0.52
J20_S18	0.12	0.88	0.61	0.61	-0.90	0.76	-0.59	2.43
J20_S19	0.02	-0.40	0.73	0.35	-0.90	0.16	-0.01	-1.21
J20_S20	0.64	0.76	2.08	-0.76	-0.90	-1.05	-1.17	-0.75
J21_Ms	-0.24	-0.27	-0.47	-1.15	0.80	-0.14	-0.59	0.79
J21_Mm	0.43	-0.59	-0.14	-0.46	2.98	-0.45	-0.59	-0.21
J21_Mn	0.95	-0.05	-0.12	0.66	1.41	-0.14	-0.01	0.07
J21_N1	-0.71	-0.06	-0.37	1.00	-0.08	-0.14	0.58	0.16
J21_N2	1.26	0.42	0.12	-0.03	0.93	-0.75	-0.01	-0.12
J21_N3	0.59	-1.60	-0.03	-0.63	1.09	-0.75	-0.59	-0.03
J21_N4	0.12	0.10	-0.35	-0.42	1.04	-0.14	-0.59	0.34
J21_N5	0.38	0.72	-0.23	-0.59	0.72	-0.14	-0.59	0.88
J21_N6	-0.19	-0.32	-0.43	-1.45	1.76	0.46	-0.59	0.61
J21_N7	-0.40	-1.21	-0.53	-1.45	0.22	0.46	-0.01	0.34
J21_N8	0.02	-1.61	-0.08	-1.66	0.61	-0.45	-0.59	-0.57
J21_N9	0.12	-1.06	-0.26	-1.62	1.04	0.76	-0.59	-0.21
J21_N10	-0.03	-1.25	-0.31	-1.23	1.92	-0.45	-0.59	-0.66
J21_N11	0.33	-0.96	-0.26	-0.93	0.16	-0.45	1.74	0.88
J21_N12	0.53	-0.59	0.00	0.18	0.19	-0.45	-0.59	3.07
J21_S1	-0.14	-0.36	-0.40	-0.85	-0.77	0.76	-0.59	0.43

For Review Only

J21_S2	-0.03	-0.60	-0.38	-0.93	-0.77	0.16	-0.59	-0.39
J21_S3	0.48	1.09	-0.19	-0.25	-0.77	-0.14	-0.59	0.70
J21_S4	0.07	0.07	-0.37	-0.59	-0.77	-0.75	-0.59	0.25
J21_S5	0.33	0.07	-0.24	-0.37	-0.77	-0.45	0.58	0.70
J21_S6	0.74	-0.27	-0.02	-0.07	-0.77	-0.14	-0.59	0.16
J21_S7	0.84	-0.22	-3.40	0.05	-0.77	0.16	-0.59	0.07
J21_S8	0.95	-0.13	0.05	0.01	-0.77	-0.45	-0.59	-0.75
J21_S9	0.07	-0.76	-0.46	0.96	-0.77	-1.05	1.74	0.61
J21_S10	0.33	-0.51	-0.33	1.00	-0.77	-1.05	-0.59	-0.12
J21_S11	-0.19	-1.29	-0.40	-1.70	-0.90	0.76	-0.59	0.52
J21_S12	0.28	-0.45	-0.26	-1.62	-0.90	-0.14	-0.59	-0.66
J21_S13	-0.14	-1.63	-0.34	-1.19	-0.90	-0.45	-0.59	-0.39
J21_S14	0.22	-1.27	-0.18	-1.19	-0.90	-0.14	-0.59	-0.48
J21_S15	-0.09	-1.53	-0.34	-1.79	-0.90	-0.45	-0.59	-0.57
J21_S16	0.53	-0.14	-0.15	-0.07	-0.90	-0.14	-0.59	-0.48
J21_S17	0.12	-0.75	-0.30	-0.72	-0.90	-0.75	-0.59	-0.12
J21_S18	0.33	-0.68	-0.28	-0.07	-0.90	-0.45	-0.59	-0.30
J21_S19	0.17	-1.36	-0.49	-0.59	-0.90	-0.45	0.58	0.43
J21_S20	0.79	0.71	-0.04	0.57	-0.90	-0.75	1.74	-0.66
N20_Mn	-2.16	1.36	-1.90	5.37	1.81	-0.45	-0.59	3.16
N20_M5	-2.41	0.74	-2.10	0.31	1.57	-0.14	-0.59	-0.75
N20_N1	-2.16	0.48	-1.93	1.64	0.32	-0.45	-0.59	0.98
N20_N2	-2.10	1.63	-1.91	-0.29	1.23	-0.14	0.58	-1.75
N20_N3	-2.21	1.26	-1.97	-0.63	1.36	-1.35	-0.59	-0.39
N20_N5	-3.91	0.67	-2.70	-0.63	1.04	1.37	-0.59	-0.30
N20_N7	-3.71	0.71	-2.64	-0.93	0.69	1.07	-0.59	0.25
N20_N9	-2.62	0.85	-2.20	1.77	1.28	1.07	-0.59	-0.94
N20_S10	-2.26	1.30	-1.93	1.64	-0.77	-0.45	-0.59	-0.75
S21_Ms	-0.24	-1.82	-0.04	-0.67	0.61	-1.05	0.58	-0.39
S21_Mn	1.10	2.64	-0.40	1.17	1.36	-0.75	-0.01	-0.39
S21_M5	0.12	-0.24	-0.16	0.48	1.44	-1.35	1.16	-0.48
S21_N1	0.95	2.78	-0.55	0.96	0.08	-0.14	0.58	-0.57
S21_N2	0.33	-0.52	-0.55	0.91	0.96	-1.35	-0.59	-0.48
S21_N5	0.07	-0.31	0.07	0.87	0.80	-1.05	0.58	-0.94
S21_N12	1.15	2.29	-0.55	1.38	0.22	-1.35	0.58	0.07
S21_S3	-0.35	-1.78	-0.06	0.18	-0.77	-1.05	0.58	-0.84
S21_S4	0.07	0.28	0.01	0.61	-0.77	-1.05	0.58	-0.94
S21_S5	-0.19	-0.66	-0.04	0.40	-0.77	-1.05	-0.59	-0.12
S21_S7	1.36	0.74	0.01	1.00	-0.77	-0.75	-0.59	-0.66
S21_S9	1.52	2.40	-0.22	1.68	-0.77	-1.35	0.58	-0.12
S21_S10	0.90	2.46	-0.42	0.96	-0.77	-0.75	0.58	-0.39
S21_S17	0.69	0.35	-0.04	0.70	-0.90	-1.05	2.91	0.07
S21_S18	0.59	-1.07	-0.25	-0.16	-0.90	-1.65	1.74	-0.48
S21_S20	1.41	2.65	0.59	0.10	-0.90	0.16	1.74	-0.21

For Review Only

analysis (RDA). Abbreviations: T,
ate; NH_4^+, ammonium; TN, total

TN (mg/L)	TP (mg/L)	Chl (µg/L)
0.30	0.69	-0.52
-0.03	-0.29	-0.50
-0.62	-0.29	-0.51
-0.78	-0.29	-0.48
-0.78	-0.78	-0.47
-0.13	-0.29	-0.58
7.06	0.69	-0.38
-0.84	0.20	-0.47
0.25	0.69	-0.44
0.46	0.20	-0.49
-0.46	-0.29	-0.49
0.46	1.18	-0.47
-0.78	-0.29	-0.58
-0.30	-0.78	-0.39
-0.40	-0.78	-0.56
-0.30	2.16	-0.60
-0.03	0.20	-0.38
-1.27	-0.29	-0.56
0.89	0.69	-0.31
0.41	0.20	-0.40
0.30	0.20	-0.35
-0.40	-0.78	-0.60
-0.46	-1.27	-0.56
-0.73	-0.78	-0.60
-0.67	0.20	-0.55
0.41	0.20	-0.44
0.57	0.20	-0.60
-0.35	-0.29	-0.58
-0.19	0.20	-0.49
1.87	2.65	-0.44
-0.24	0.20	-0.49
-1.38	-0.29	-0.58
-1.11	-0.78	-0.40
-0.13	-0.78	-0.48
-1.05	-1.27	-0.62
0.25	-0.29	0.05
-0.08	-0.78	-0.32
0.03	-0.78	-0.49
-1.11	0.20	-0.19
-0.13	-0.78	-0.22
0.19	-0.29	-0.03
0.35	-0.78	0.07
0.25	-0.29	-0.27
0.35	-0.78	-0.02
0.08	-0.29	0.02
-0.24	-0.78	-0.22
0.46	-0.78	-0.28
0.19	-0.78	-0.36
-0.73	-0.29	-0.28
-0.40	-0.29	-0.27
0.14	-0.78	-0.08

For Review Only

0.62	-0.29	0.04
0.73	-0.29	-0.27
0.52	-0.78	-0.05
0.19	-0.29	0.21
0.03	-0.78	-0.29
-0.19	-0.78	-0.45
-0.19	-0.29	-0.19
-0.40	-0.78	-0.34
-0.08	-0.29	-0.36
0.14	-0.29	-0.17
-0.35	-0.78	-0.08
-0.13	-0.78	-0.36
0.14	-0.78	-0.34
0.35	0.20	0.30
0.41	-1.27	-0.55
0.14	-0.78	-0.45
0.14	-0.78	-0.29
-0.51	0.20	-0.01
-0.84	-0.78	-0.08
0.79	0.69	0.10
-0.35	-0.78	-0.06
2.90	2.16	0.23
-0.08	-0.29	0.16
0.25	-0.29	0.37
-0.19	-0.29	0.02
-0.67	-1.27	-0.27
0.08	-0.29	-0.17
0.03	0.20	0.44
-1.00	0.20	1.49
-0.62	-0.78	0.20
0.52	1.18	1.66
0.03	0.69	6.55
1.44	3.63	2.93
-0.89	1.18	0.91
0.41	2.16	2.45
-1.22	0.20	1.20
-1.11	0.69	0.85
1.71	4.12	3.64
0.25	1.67	-0.38
-0.89	1.18	-0.45
-0.67	-0.78	0.61
-0.24	1.18	0.26
0.35	1.18	1.58
-0.67	1.18	-0.31

For Review Only

Table S3. Pearson correlation matrix (upper triangle) and variance inflation factors (VIFs) for the 11 en
correlations (r) are shown above the diagonal; blank cells indicate the lower triangle omitted for clarity.
Thresholds used: r ≥ 0.7 and/or VIF ≥ 5 indicate potential collinearity.

Variable	T	DO	Con	pH	Dep	NO_3^-	PO_3^-	NH_4^+
T	1	0	0.59	-0.03	-0.27	-0.14	0.17	-0.07
DO		1	-0.16	0.54	0.09	-0.05	0.03	-0.04
Con			1	-0.13	-0.21	0.22	0.15	0.01
pH				1	0.1	-0.15	0.04	0.07
Dep					1	-0.03	-0.13	0.02
NO3						1	0.12	0.21
PO3							1	0.05
NH4								1
TN								
TP								
Chl								

For Review Only

ıvironmental variables used in RDA. Pearson
. VIF values are listed in the last column.

TN	TP	Chl-a	VIF
-0.11	-0.01	0.02	1.95
-0.1	0.07	0.19	1.52
-0.07	0.02	-0.25	2.06
-0.01	0.27	0.19	1.6
0.13	-0.05	0.06	1.14
0.05	-0.07	-0.34	1.39
0.02	0.27	0.1	1.16
0.05	0.08	-0.13	1.09
1	0.38	0.12	1.27
	1	0.49	1.93
		1	1.73

For Review Only

Table S4. Number of clean sequence reads of fish taxa at each site.

**Taxa**	**Jun20_M1**	**Jun20_M1**	**Jun20_M1**	**Jun20_M2**	**Jun20_M2**	**Jun20_M2**
Acheilognathus chankaensis	0	0	0	0	0	0
Carassius auratus	81256	8400	50761	14406	26282	58062
Channa argus	0	0	0	0	0	0
Clarias fuscus	0	0	0	0	0	0
Ctenopharyngodon idella	0	0	0	0	0	0
Cyprininae	9837	3	7	4561	133	7668
Cyprinus carpio	17627	11	145	200	10857	45683
Gambusia affinis	0	0	0	0	0	0
Hemiculter leucisculus	2	0	0	0	1	0
Homatula spp.	0	0	0	0	0	0
Hypomesus olidus	71896	37101	107748	34981	42352	18445
Hypophthalmichthys spp.	62154	87538	65894	32100	364	28332
Hypophthalmichthys molitrix	16433	72342	52542	11521	17667	7520
Hypophthalmichthys nobilis	54	2979	93	373	0	25
Megalobrama amblycephala	0	0	0	0	0	0
Micropercops swinhonis	42	4	11	10	11	11
Micropterus salmoides	4	1	4	1	3	0
Misgurnus spp.	0	0	0	0	0	0
Misgurnus anguillicaudatus	0	0	0	0	0	0
Misgurnus dabryanus	0	0	0	0	0	0
Monopterus albus	0	0	0	0	0	0
Mylopharyngodon piceus	0	0	0	0	0	0
Neosalanx taihuensis	47	25	37	23	25139	30
Oryzias latipes	0	0	0	0	0	0
Pseudorasbora parva	4	3	3	2	29324	2
Rhinogobius spp.	0	0	0	0	0	9
Rhinogobius cliffordpopei	4	0	2	1	4	13679
Rhinogobius giurinus	11	20272	14	55192	27978	33675
Rhodeus spp.	3	0	9	11	0	4
Rhodeus ocellatus	0	0	0	0	0	0
Rhodeus sinensis	9085	6	9436	7836	6	3618
Schizothorax spp.	0	0	0	0	0	0
Schizothorax griseus	0	0	0	0	0	0
Schizothorax taliensis	0	0	0	0	0	0
Silurus spp.	0	0	0	0	0	0
Siniperca	0	0	0	0	0	0
Squaliobarbus curriculus	0	0	0	0	0	0
Tachysurus fulvidraco	5	1	2	1	2	5
Unclassifies	106746	253	23	13	14	3683

For Review Only

Jun20_M3	Jun20_M3	Jun20_M3	Jun20_M4	Jun20_M4	Jun20_M4	Jun20_M5	Jun20_M5	Jun20_M5
0	0	0	0	0	0	0	0	0
23720	22693	35083	1998	48579	69813	136	146274	57020
0	0	0	0	0	0	0	0	0
0	0	0	0	0	0	0	0	0
0	0	0	0	0	0	0	0	0
7	14	11	10	4	11	2	10	14
60	37	54	659	96	102	10	258	95
0	0	0	0	0	0	0	0	0
5078	1	0	1	1	7041	3	1	3
0	0	0	0	0	0	0	0	0
22023	38	39823	28	33984	38	52	24	36655
9726	33	14218	978	10701	581	112201	41210	14787
6421	18	12509	29	17382	29598	1493	453	150
3	0	9	0	13	1	58	12	3
0	0	0	0	0	0	0	0	0
6	7	7	475	32727	7	14	9	13
1	2	1	2	0	3	1	2	1
0	0	0	326	0	0	0	0	0
0	0	0	0	0	0	0	0	0
0	0	0	2	0	0	0	0	0
0	0	0	0	0	0	0	0	0
0	0	0	0	0	0	0	0	0
29	158032	18	1126	38462	21265	86	61932	89381
0	0	0	0	0	0	0	0	0
4841	4	2	1153	4	6424	3	2	6
0	0	0	0	0	0	0	0	0
1	0	0	0	1	1	1	1	1
9317	18	12	1373	10	7594	23	9	11515
0	0	0	0	29	0	0	0	0
0	0	0	0	0	0	0	0	0
2	6	6	1	24804	2	1	3	4
0	0	0	0	0	0	0	0	0
0	0	0	0	0	0	0	0	0
0	0	0	0	0	0	0	0	0
0	0	0	0	0	0	0	0	0
0	0	0	0	0	0	0	0	0
0	0	0	0	0	0	0	0	0
3	7	7	446	2	4	17	3	12094
4	20416	4	296	6904	9	11	1900	1452

For Review Only

Jun20_M6	Jun20_M6	Jun20_M6	Jun20_M7	Jun20_M7	Jun20_M7	Jun20_M8	Jun20_M8	Jun20_M8
0	0	0	0	0	0	0	0	0
53231	183663	24088	227340	260765	268223	165789	282485	165753
0	0	0	0	0	0	0	0	0
0	0	0	0	0	0	0	0	0
0	0	0	0	0	0	0	0	0
6079	83	5	21	29	24	17	17	30
13647	361	56	309	268	513	226	417	285
0	0	0	0	0	0	1	0	0
0	1	0	4	1	1	0	1	0
0	0	0	0	0	0	0	0	0
27099	23274	45	27	41	9	3	57527	5681
48932	56192	51718	183	12022	37913	77057	14923	2701
456	801	23778	13416	6449	15779	23042	34447	21
25	46	23	1	0	19	24	5	1
0	0	0	0	0	0	0	0	0
86993	14461	12	11	4	1	0	0	0
2	6077	17135	2	4	0	2	2	1
0	0	0	0	0	0	0	0	1
0	0	0	0	0	0	0	0	0
0	0	0	0	0	2	2	2	1
0	0	0	0	0	0	0	0	0
0	0	0	0	0	0	0	0	0
58058	37772	13129	22	2626	0	0	0	0
0	0	0	0	0	0	0	0	0
3	6969	4	1	3	2	0	2	0
0	0	0	0	0	0	0	0	0
0	0	3	0	1	0	0	0	0
13656	16	21667	8049	14	1	0	2	1
0	0	0	0	0	0	0	0	0
0	0	0	0	0	0	0	0	0
4	4	3	4	4	0	0	0	0
0	0	0	0	0	0	0	0	0
0	0	0	0	0	0	0	0	0
0	0	0	0	0	0	0	0	0
0	0	0	0	0	0	0	0	0
0	0	0	0	0	0	0	0	0
0	0	0	0	0	0	0	0	0
10843	5659	21728	2	6	1	2	2	3
7056	730	15	17	19	13	7	18	2

For Review Only

Jun20_M9	Jun20_M9	Jun20_M9	Jun20_M1	Jun20_M1	Jun20_M1	Jun20_M1	Jun20_M1	Jun20_M1
0	0	0	0	0	0	0	0	0
38471	55296	21886	43001	45452	50864	26750	14585	16079
0	0	0	0	0	0	0	0	0
0	0	0	0	0	0	0	0	0
0	0	0	0	0	0	0	0	0
16479	10	3	4	150	5	0	2	100
1592	102	47	161	14494	263	136	24	11512
0	0	0	0	0	0	0	0	0
23133	0	3	1	3	2	3	2	2
0	0	0	0	0	0	0	0	0
9	1	68258	8	10	8	16819	5	8
36462	3570	75955	21962	22103	41141	44	12377	3565
24593	36	10058	18883	25160	27963	20	3762	2310
77	1	19	2	1	84	0	4	0
0	0	0	0	0	0	0	0	0
28231	1	0	0	0	0	2	0	2
3693	58552	6	5	6428	2757	25	8207	4
29949	1	1	1	0	0	1	6	0
0	0	0	0	0	0	0	0	0
152	1	2	3	0	3	4	2279	1
0	0	0	0	0	0	0	0	0
0	0	0	0	0	0	0	0	0
1	1	2	0	0	0	0	1	7666
0	0	0	0	0	0	0	0	0
57094	2	2	2	0	1	0	2	18065
0	0	0	0	0	0	0	0	0
0	0	0	0	0	0	0	0	0
1	2	1	1	1	2	2	2	32956
0	0	0	0	0	0	0	0	0
0	0	0	0	0	0	0	0	0
0	0	0	0	67	0	0	0	0
0	0	0	0	0	0	0	0	0
0	0	0	0	0	0	0	0	0
0	0	0	0	0	0	0	0	0
0	0	0	0	0	0	0	0	0
0	0	0	0	0	0	0	0	0
0	0	0	0	0	0	0	0	0
3	2	2	4	1	4	22	8036	3
27016	5	75	23945	39618	9	10	456	55

For Review Only

**Jun20_M1**	**Jun20_M1**	**Jun20_M1**	**Jun20_N1-**	**Jun20_N1-**	**Jun20_N1-**	**Jun20_N2-**	**Jun20_N2-**	**Jun20_N2-**
0	0	0	0	0	0	0	0	0
569	38	3502	126032	89447	718	9711	44	32276
0	0	0	0	0	0	0	0	0
0	0	0	0	0	0	0	0	0
0	0	0	0	0	0	0	0	0
17	0	4540	6	67	3	59	0	3
1054	2	486	183	5329	3	6550	2	54
0	0	0	0	1564	0	1	0	3
0	3	0	5003	0	12463	1	1	2
0	0	0	0	0	0	0	0	0
20	5191	6	11	40076	7	13	15209	22471
118	8963	10	8	11	9	13	10	244
8	101	0	4	10	6	7	3	8380
0	1	0	0	0	0	0	0	0
0	0	0	0	0	0	0	0	0
0	0	0	3	0	1	1	1	2
1	0	1	5	4	1	1	2	0
2	0	2	1473	210	2	1	0	0
0	0	0	0	0	0	0	0	0
0	0	0	5	24155	1	2	2	2
0	0	0	0	0	0	0	0	0
0	0	0	0	0	0	0	0	0
0	0	6833	0	0	0	0	0	3
0	0	0	0	0	0	0	0	0
8	4094	2	1	2	3	3	3971	0
0	0	0	0	0	0	0	0	0
0	0	0	0	0	0	0	0	1
592	0	15488	0	3	1	2	0	41
0	0	0	0	0	0	0	0	0
0	0	0	0	0	0	0	0	0
2	0	0	0	0	0	0	0	2
0	0	0	0	0	0	0	0	0
0	0	0	0	0	0	0	0	0
0	0	0	0	0	0	0	0	0
0	0	0	0	0	0	0	0	0
0	0	0	0	0	0	0	0	0
0	0	0	0	0	0	0	0	0
122	3	2	2563	29486	0	1	2	0
2380	38464	7	12	10	234253	4	13559	2

For Review Only

Jun20_N3·	Jun20_N3·	Jun20_N3·	Jun20_N4·	Jun20_N4·	Jun20_N4·	Jun20_N5·	Jun20_N5·	Jun20_N5·
0	0	0	0	0	0	0	0	0
64494	734	37740	61745	36668	37925	64717	56385	84158
0	0	0	0	0	0	0	0	0
0	0	0	0	0	0	0	0	0
0	0	2	0	0	2	0	0	0
420	460	220	101	156	5	16	6	8
45164	50181	32321	4570	17642	109	254	122	165
56699	0	1	0	1	0	1	2	0
1	1	3	14628	2	20764	24	10018	14727
0	0	0	0	0	0	0	0	0
2	2	6	17846	4	60308	6686	38148	12
154	38493	16	7611	5429	129715	121	19	13
1592	536	1	29105	47	6037	8	2	1
2	14	0	3	2	104	1	0	0
0	0	0	0	0	0	0	0	0
1	0	11306	1	1	1	4	33416	22180
0	0	0	0	0	0	0	0	0
5857	0	1	1	158	2	1	3	1
0	0	0	0	0	0	0	0	0
62405	1	0	1	13807	4	2	1	1
0	0	0	0	0	0	0	0	0
0	0	0	0	0	0	0	0	0
4	40682	9	78917	18517	6337	3	2	1
0	0	0	0	0	0	0	0	0
5	2	0	1	1	1	24733	1	7330
0	0	0	0	0	0	0	0	2
2	0	1	2	0	1	7	0	25660
8832	22708	3427	80063	72836	22497	9254	4	7
0	0	0	0	0	0	0	0	0
0	0	0	0	0	0	0	0	0
0	1	2	0	2	1	1	2	1
0	0	0	0	0	0	0	0	0
0	0	0	0	0	0	0	0	0
0	0	0	0	0	0	0	0	0
0	0	0	0	0	0	0	0	0
0	0	0	0	0	0	0	0	0
0	0	0	0	0	0	0	0	0
0	0	0	0	0	0	0	0	0
0	1	0	12035	0	0	0	0	2
7552	6	16739	1	1	0	21309	11546	10560

For Review Only

Jun20_N6-	Jun20_N6-	Jun20_N6-	Jun20_N7-	Jun20_N7-	Jun20_N7-	Jun20_N8-	Jun20_N8-	Jun20_N8-
0	0	0	0	0	0	0	0	0
120638	209029	196458	10002	25131	10701	2929	32520	2577
0	0	0	0	0	0	0	0	0
0	0	0	0	0	0	0	0	0
0	0	0	1	0	0	1	0	0
16	77	12	167	1769	132	81	126	0
248	6056	382	16297	4518	8943	7372	10851	14
0	1	2	0	0	0	0	0	1
4	6913	3	20447	6057	13	2	3	2
0	0	0	0	0	0	0	0	0
13938	8454	6	29543	22435	55858	3567	6	3
3792	6456	4949	22543	12785	35516	3248	4	1276
12644	2765	5230	3991	4775	19636	4184	3	1223
10	0	4	7	6	42	5	0	0
0	0	0	0	0	0	0	0	0
5592	1	0	2	7655	10206	0	2	2
0	0	0	0	0	0	0	0	0
107	12766	0	0	3094	7556	2	1	471
0	0	0	0	0	0	0	0	0
691	9500	0	1	139	48	0	1	61
0	0	0	0	0	0	0	0	0
0	0	0	0	0	0	0	0	0
2	6	4	2	19651	27126	6147	2	2458
0	0	0	0	0	0	0	0	0
1	3	2	8345	6114	6712	1	6480	1734
0	0	0	1	0	0	0	0	0
2	0	1	21361	0	8854	0	1	3
9391	14168	4	28826	4	29344	2639	11226	2
6	0	0	32	0	11	0	0	0
0	0	0	0	0	0	0	0	0
8983	0	265	8218	2	13599	0	0	1
0	0	0	0	0	0	0	0	0
0	0	0	0	0	0	0	0	0
0	0	0	0	0	0	0	0	0
0	0	0	0	0	0	0	0	0
0	0	0	0	0	0	0	0	1551
0	0	0	0	0	0	0	0	1331
1	2	0	2	0	0	25685	1	1
0	709	1	1	26	2	1	5919	43154

For Review Only

**Jun20_N9**	**Jun20_N9**	**Jun20_N9**	**Jun20_N1**	**Jun20_N1**	**Jun20_N1**	**Jun20_N1**	**Jun20_N1**	**Jun20_N1**
0	0	0	0	0	0	0	0	0
23613	7	836	2933	2478	1324	8258	1544	5764
0	0	0	0	0	0	0	0	0
0	0	0	0	0	0	0	0	0
18501	0	0	0	0	0	0	0	0
0	3	0	4	1153	0	1564	0	1
36	603	1	1070	722	5	131	4	10
0	0	0	0	0	0	0	0	2
4	1	1585	0	1	511	463	1	0
0	0	0	0	0	0	0	0	0
10133	628	1	1629	838	3	688	1	2866
8	0	3	89	0	0	1	0	1
1	0	0	1078	2	0	0	0	0
0	0	0	3	0	0	0	0	0
0	0	0	0	0	0	0	0	0
2	1	0	0	0	21	0	0	1
0	0	0	0	0	0	0	0	0
0	0	4	0	0	0	0	0	0
0	0	0	0	0	0	0	0	0
1	0	983	0	0	0	1	0	0
0	0	0	0	0	0	0	0	0
0	0	0	0	0	0	0	0	0
22179	0	14212	0	684	0	0	0	2019
0	0	0	0	0	0	0	0	0
2	1832	3	777	3403	5018	2	1	1881
0	0	0	0	0	0	0	0	0
0	0	0	0	0	1	0	0	0
29522	887	4805	951	1619	1206	788	0	5544
8	0	0	0	0	0	0	0	0
0	0	0	0	0	0	0	0	0
5226	423	0	1245	0	0	0	0	0
0	0	0	0	0	0	0	0	0
0	0	0	0	0	0	0	0	0
0	0	0	0	0	0	0	0	0
0	0	0	0	0	0	0	0	0
0	0	0	0	0	0	0	0	0
0	0	0	0	0	0	0	0	0
1	0	0	0	1	0	0	0	0
2	542	531	594	2	0	0	58	0

For Review Only

Jun20_N1.	Jun20_N1.	Jun20_N1.	Jun20_S1-	Jun20_S1-	Jun20_S1-	Jun20_S2-	Jun20_S2-	Jun20_S2-
0	0	0	0	0	0	0	0	0
2812	3159	1312	12042	18851	19454	16729	21768	20300
0	0	0	0	0	746	0	0	0
0	0	0	0	0	0	0	0	0
0	0	0	0	0	0	0	0	0
0	8	3	1	15	21	37	103	58
3	1185	425	353	1203	1400	2579	6708	3511
788	0	0	0	0	0	0	0	0
526	491	0	1124	2386	3297	8700	9568	5016
0	0	0	0	0	0	0	0	0
319	1	0	1161	387	611	1454	1415	1067
658	0	1	9	1	0	1063	1264	243
7	0	0	682	0	0	5939	2999	1933
0	0	0	0	0	0	5	0	0
0	0	0	0	0	0	0	0	0
551	408	758	2553	4985	6751	2415	513	377
875	0	0	0	0	0	0	0	0
0	0	0	2343	6580	7061	0	623	16
0	0	0	49	0	2122	0	0	542
0	0	0	1096	5853	5964	0	4	674
0	0	0	0	0	0	0	0	0
0	0	0	0	0	0	0	0	0
278	0	2466	0	0	0	0	0	0
0	0	0	0	0	0	0	0	0
2556	1888	305	7252	2334	2574	9372	7733	9219
0	0	0	283	303	2	0	0	418
0	0	533	11094	6566	5294	1126	1	2752
630	0	625	341	1835	1011	1070	492	612
0	0	0	2	0	3	1	4	6
0	0	0	0	0	0	0	0	0
0	0	0	4537	2701	1619	2835	5559	1868
0	0	0	0	0	0	0	0	0
0	0	0	0	0	0	0	0	0
0	0	0	0	0	0	0	0	0
0	0	0	1079	148	691	1149	1799	1
0	0	0	0	0	0	0	0	0
0	0	0	0	0	0	0	0	0
0	0	0	2846	1971	1182	0	0	734
0	0	5898	875	0	0	0	331	941

For Review Only

Jun20_S3-	Jun20_S3-	Jun20_S3-	Jun20_S4-	Jun20_S4-	Jun20_S4-	Jun20_S5-	Jun20_S5-	Jun20_S5-
0	0	0	0	0	0	0	0	0
31282	36819	17353	212458	117682	166090	15857	11965	4099
0	0	0	0	2	5080	0	2	1
0	0	0	0	0	0	0	0	0
0	0	0	0	0	0	0	0	0
11	25	6	32	6	28	29	52	21
1069	1196	1093	1901	218	1862	2615	5069	1878
0	0	0	1531	0	0	0	0	0
881	2705	6724	2615	2315	62	37551	50159	17838
0	0	0	0	0	0	0	0	0
0	0	0	840	2077	3	3783	5584	5452
1	1	1	2	0	2	251	8939	931
2	0	0	2	0	1	1864	9409	2091
0	0	0	0	0	0	5	4	7
0	0	0	0	0	0	0	0	0
1228	510	1041	35651	34743	36775	12687	21892	6421
0	0	0	0	0	0	0	0	0
16	491	5	0	1299	1	0	1	0
0	0	0	0	0	0	0	0	1
1058	2	918	1	9	1	0	0	0
0	0	0	0	0	0	0	0	0
0	0	0	0	0	0	0	0	0
0	0	0	0	0	0	559	1	727
0	0	0	0	0	0	0	0	0
2419	2456	3090	44574	83777	99313	78587	69326	17628
0	0	0	10	1056	7	9	9	1
0	0	1141	37333	36358	75131	29742	39782	5034
0	495	493	46521	41777	35974	102253	148745	360658
3	14	14	3	3	0	1	0	0
0	0	0	0	0	0	0	0	0
5146	16422	15777	1242	3093	2	2840	6	4
0	0	0	0	0	0	0	0	0
0	0	0	0	0	0	0	0	0
0	0	0	0	0	0	0	0	0
0	0	0	0	1279	0	0	0	1
0	0	0	0	0	0	0	0	0
0	0	0	0	0	0	0	0	0
0	161	1089	2150	2799	1	1	2635	1
204	302	0	8898	0	1297	29	2	639

For Review Only

Jun20_S6-	Jun20_S6-	Jun20_S6-	Jun20_S7-	Jun20_S7-	Jun20_S7-	Jun20_S8-	Jun20_S8-	Jun20_S8-
0	0	0	0	0	0	0	0	0
99998	61383	76660	3840	7245	3353	7134	16190	2084
0	15976	2	3393	2	3406	349	2	1
0	0	0	0	0	0	0	0	0
0	0	0	0	0	0	0	0	0
7	9	13	142	154	25	2869	1053	27
213	146	149	12445	13922	2497	9427	20241	2421
0	0	0	0	0	0	0	0	0
38910	42326	34078	244032	267534	163105	23492	38782	42181
0	0	0	0	0	0	0	0	0
10142	4	5716	961	3	5397	3	3	1
3	98	4	0	0	47	1228	283	0
1	3632	3	2	0	783	426	6	2
0	0	0	0	0	0	0	0	0
0	0	0	0	0	0	0	0	0
21412	11176	8188	4122	13	5	1412	8	10
0	0	0	0	0	0	0	0	0
0	3	1	14	2	0	0	3	0
0	0	0	0	0	0	0	0	0
0	2254	0	914	0	0	0	260	0
0	0	0	0	0	0	0	0	0
0	0	0	0	0	0	0	0	0
0	0	0	1547	0	0	0	1	0
0	0	0	0	0	0	0	0	0
53323	26105	67972	3537	3473	3711	2945	2688	4511
7	7	4	8	1	2	4	2	6
51460	58871	34745	47937	15111	5584	5001	18107	10682
100492	117488	80462	1506	11316	7702	4963	4374	5875
0	15	2	0	1	0	5	7	12
0	0	0	0	0	0	0	0	0
7	17275	6934	3966	3392	3	8646	11724	10992
0	0	0	0	0	0	0	0	0
0	0	0	0	0	0	0	0	0
0	0	0	0	0	0	0	0	0
0	0	1	0	0	0	0	0	0
0	0	0	0	0	0	0	0	0
0	0	0	0	0	0	0	0	0
1	1	3	1	0	1	0	1983	2235
1793	5326	3	2	4636	1797	1	782	612

For Review Only

**Jun20_S9-**	**Jun20_S9-**	**Jun20_S9-**	**Jun20_S10**	**Jun20_S10**	**Jun20_S10**	**Jun20_S11**	**Jun20_S11**	**Jun20_S11**
0	0	0	0	0	0	0	0	0
2802	4855	4647	27768	66361	44513	44672	79468	110368
0	1	0	2	1442	531	0	0	1
0	0	0	0	0	0	0	0	0
14	0	0	0	0	0	4	1358	670
17	2	1	26	68	14	97	63	80
1224	6	16	2276	5961	884	4185	5691	9150
0	0	0	0	0	1	0	0	310
51891	45141	75377	43714	103055	47078	3519	1396	3244
0	0	0	0	0	0	0	0	0
829	1	1	1	2	2	0	0	805
3	2	6050	6	1	6	4957	4905	0
3	0	181	1	3	1	61	68	1
0	0	4	0	0	0	2	2	0
0	0	0	0	0	0	0	0	0
5	5	1947	5	873	1521	33664	48801	59042
0	0	0	0	0	0	0	0	2
4433	7154	9521	835	1895	772	17455	13593	19707
0	0	4034	825	0	2289	0	0	1224
21	78	36	4	12	1	5265	1219	3221
0	0	0	0	0	0	0	0	0
0	0	0	0	0	0	0	0	0
0	0	20	0	0	0	0	0	0
0	0	0	0	0	0	0	0	0
2732	18	5003	1873	5469	6267	14298	14519	7215
2	2	11	12	6649	487	2	3	2
9266	5779	20394	23248	43979	17375	13388	14864	24184
6284	11046	3859	39	2570	678	2546	6271	3381
1	3	0	6	11	26	0	0	8
0	0	0	1	0	0	0	0	227
939	5808	6	5481	15049	7709	10	6	1158
0	0	0	0	0	0	0	0	0
0	0	0	0	0	0	0	0	0
0	0	0	0	0	0	0	0	0
0	0	0	0	0	0	3	2	0
0	0	0	0	0	0	0	0	0
677	0	0	0	0	0	0	0	0
2119	2530	1957	1635	3279	1376	1472	43387	8415
0	4	3	53	26	3485	482	0	7

For Review Only

**Jun20_S12**	**Jun20_S12**	**Jun20_S12**	**Jun20_S13**	**Jun20_S13**	**Jun20_S13**	**Jun20_S14**	**Jun20_S14**	**Jun20_S14**
0	0	0	0	0	0	0	0	0
146963	135391	100141	99355	76240	99799	21459	27381	8749
1295	1390	1332	118	0	0	1	0	461
0	0	0	0	0	0	0	0	0
1	0	0	0	723	535	1163	1004	598
73	79	107	3082	1523	2149	4531	4982	927
5252	6528	7559	40863	50035	50390	41067	96806	17462
3	777	311	2	0	1	3636	188	2600
14686	20404	16282	105980	132206	93579	204901	201919	137961
0	0	0	0	0	0	0	0	0
0	0	929	3199	0	1339	1	6307	1741
1	0	0	0	0	0	0	1	0
0	1	0	0	0	0	1	124	0
0	0	0	0	0	0	0	0	0
0	0	0	0	0	0	0	0	0
20462	9420	8502	4168	5056	5823	25031	11403	10093
2	708	0	0	0	0	0	0	0
26548	605	4053	1115	2	0	3972	446	1976
1	1	0	0	1	0	1	1	393
1339	2389	493	6	0	0	1932	137	795
0	0	0	0	0	0	0	0	0
0	0	0	0	0	0	0	0	0
1349	0	1	6	1214	0	737	3800	2109
0	0	0	0	0	0	0	0	0
26505	11695	7452	3038	5083	9325	6278	3851	3275
1	1	2	6	6	3547	4330	7	889
13784	16905	10373	17507	23184	15725	21443	18187	63701
2	637	3580	12256	10353	1795	4111	2679	8563
32	60	56	12	7	7	51	48	23
1	1	0	0	0	0	0	0	0
53966	51776	59488	13584	6051	5741	34725	37941	24792
0	0	0	0	0	0	0	0	0
0	0	0	0	0	0	0	0	0
0	0	0	0	0	0	0	0	0
8	586	328	18704	9562	22224	1216	2856	1252
0	0	0	0	0	0	0	0	0
0	0	0	0	0	0	0	0	0
2188	4222	4205	463	3106	2404	1275	993	400
2	90	560	370	2643	2512	6	0	0

For Review Only

Jun20_S15	Jun20_S15	Jun20_S15	Jun20_S16	Jun20_S16	Jun20_S16	Jun20_S17	Jun20_S17	Jun20_S17
0	0	0	0	0	0	0	0	0
90619	109439	83092	51541	30910	26999	213962	133202	224657
0	0	0	0	0	0	0	0	0
0	0	0	0	0	0	0	0	0
0	0	0	0	0	0	0	0	0
110	24	589	9746	4052	2987	26	2138	7245
5217	2731	1943	11609	10800	15268	462	3030	1153
6299	7124	8473	0	2	2	8115	3	0
36153	47914	37330	191217	122063	110624	12621	52603	24004
0	0	0	0	0	0	0	0	0
0	0	0	0	0	0	0	1	0
0	0	0	1	1961	0	0	0	2
0	0	0	1	27	0	0	1	2
0	0	0	0	1	0	0	0	0
0	0	0	0	0	0	0	0	0
26271	53763	54604	969	14	8	19835	31062	28749
0	0	0	0	0	0	0	0	0
15003	25177	20468	921	0	0	5003	25509	7316
10449	19335	12448	1	0	0	14830	8615	1
2115	5332	4739	985	0	0	28	2636	24
0	0	0	0	0	0	0	0	0
0	0	0	0	0	0	0	0	0
0	0	0	0	1	1	0	0	0
0	0	0	0	0	0	0	0	0
1864	6808	3649	3811	5957	4255	2941	3245	12
2	3	5	1401	8	3016	4233	3919	6
11499	15654	17115	31111	21805	16661	31003	82650	80113
5468	12721	4322	30899	15793	16342	8016	5392	13
24	25	62	55	9	29	53	15	33
1	0	0	0	0	0	0	0	0
29236	26053	57087	58800	11725	29490	65230	25176	31598
0	0	0	0	0	0	0	0	0
0	0	0	0	0	0	0	0	0
0	0	0	0	0	0	0	0	0
1579	4822	3065	1131	1	0	10953	1961	0
0	0	0	0	0	0	0	0	0
0	0	0	0	0	0	0	0	0
0	1	1	2095	5015	3442	2	1	4
0	247	1	520	1	0	0	0	15

For Review Only

Jun20_S18	Jun20_S18	Jun20_S18	Jun20_S19	Jun20_S19	Jun20_S19	Jun20_S20	Jun20_S20	Jun20_S20
0	0	0	0	0	0	0	0	0
92674	96885	70464	37633	36008	117848	24337	19706	19756
0	1940	0	2064	0	0	0	0	0
0	0	0	0	0	0	0	0	0
0	0	0	0	0	0	0	0	0
36	74	85	117	40	126	678	1069	373
3116	5484	8318	6273	4118	7556	57401	91069	31805
6	0	2	1	1	4	10835	6910	7240
10511	13614	6432	193557	110221	119048	85019	85830	94230
0	0	0	0	0	0	0	0	0
2	1	1	2784	4776	0	0	0	0
0	1	0	0	1	1	0	1	391
1	0	0	0	0	1	0	0	0
0	0	0	0	0	0	0	0	0
0	0	0	0	0	0	0	0	0
33221	68104	72403	28	13	1848	49505	47471	72353
0	0	0	1	0	0	3	1	773
9	531	2	3	3	1185	2577	45	2507
1	0	0	0	1	1	3	957	0
0	6	0	0	0	959	590	1006	13
0	0	0	0	0	0	0	0	0
0	0	0	0	0	0	0	0	0
1	3	6	19776	3521	2	2	0	0
0	0	0	0	0	0	0	0	0
14804	1230	4189	6701	3804	7600	29239	21082	24742
5	3231	2	5600	3	6	13	2875	975
34313	44705	3018	37350	18595	28106	51510	27991	61291
5840	29684	8033	69295	64671	35349	12961	8098	8172
122	117	101	8	4	30	76	73	110
0	0	1	0	0	0	0	0	0
111329	110207	105738	11265	5470	25781	90733	88199	96963
0	0	0	0	0	0	0	0	0
0	0	0	0	0	0	0	0	0
0	0	0	0	0	0	0	0	0
0	0	0	1	0	1	1	1	1
0	0	0	0	0	0	0	0	0
0	0	0	0	0	0	0	0	0
4	2452	1769	14593	19541	31174	403	1910	1340
2	1421	2676	0	11	71	99	454	6

For Review Only

Nov20_M1	Nov20_M1	Nov20_M1	Nov20_M1	Nov20_M5	Nov20_N1	Nov20_N1	Nov20_N1	Nov20_N2
46	14169	1	0	6	0	0	0	0
14476	129339	72454	73012	404	31264	72932	116797	9529
328	87212	4	6	30	1	0	1	0
0	0	0	0	0	0	0	0	0
2	1	5	2	3	16	83	19	14
49	9895	3210	3189	14	1116	4746	6753	317
12	155	14443	179	4	25	62	1158	12
5	5	4	10	7	1	1	0	0
9208	64	44	54	106	80085	94935	121889	98
0	0	0	0	0	0	0	0	0
67488	75	32692	20638	419	76991	52774	38880	63708
1	6	78	19631	4	14	44	101	27917
5	18	8388	11111	11	46	18076	7	48
0	0	2	1	0	0	0	0	111
0	0	4	0	0	7	11	25	1
9	7	12	10	17	106	50006	83614	7
0	0	0	0	0	0	0	0	0
1	0	14	3265	2	7	11	13	5
1	1	0	1	0	0	0	0	0
0	0	2	0	0	0	0	0	0
0	0	0	0	0	0	0	0	0
0	0	0	0	0	0	0	0	0
5144	17	8804	2	26	10044	5	3	8
0	0	0	0	0	0	0	0	0
8	9	29	4367	11	35025	15819	36643	2
0	0	0	0	1	0	24	19	0
7	8	9	14	11	68	33069	42294	6
8337	34951	53427	5927	340	0	49	31764	9883
0	0	0	0	0	0	0	0	0
0	0	0	0	0	0	0	0	0
2	2	6	9	9	5	1	1	1
0	0	0	0	0	0	0	0	0
0	0	0	0	0	0	0	0	0
0	0	0	0	0	0	0	0	0
1	0	0	0	0	0	0	1	0
1	0	5	0	0	17	13	58	43
0	0	5	1	1	16	8	77	32
12	5	7	11	7	1	2	1	3
9276	19492	56	10639	653	28178	29711	35062	1664

For Review Only

Nov20_N3	Nov20_N3	Nov20_N4	Nov20_N4	Nov20_N4	Nov20_N5	Nov20_N5	Nov20_N5	Nov20_N6
0	0	0	0	0	0	0	0	0
8992	6880	13343	56	7221	22205	6526	20980	16981
0	3	8	4206	0	1	0	0	0
0	0	0	0	0	0	0	0	0
35779	19609	35915	18242	25053	14940	28582	30578	23763
16	32	15	6	519	28	346	416	110
483	536	14	22	3035	12	8708	11128	9
4	5	1	1	5	6	0	0	0
16584	16815	10520	34721	1927	20211	7237	21529	19341
0	0	0	0	0	0	0	0	0
23	22	2587	1474	2701	14744	36044	22129	6273
1153	2332	15	10	5	13	16	56	22528
89	87	52	13	5	18	34	47	7003
77	93	65	71	33	9	14	18	1834
15501	14880	18901	26950	11534	25592	11127	5176	14083
7	9	4	1	1	2522	1	0	1
0	0	0	0	0	0	0	0	0
13727	10499	9399	2696	20951	17007	9747	22	12165
0	0	0	0	0	0	0	0	0
85	212	14	0	46	22	21	0	43
0	0	0	0	0	0	0	0	0
1	0	0	0	0	0	0	15	0
18325	9793	8100	2904	24205	17307	27954	26190	17237
0	0	0	0	0	0	0	0	0
2	1	1	16	1526	0	0	0	0
2	0	0	0	0	0	0	0	0
501	12	6	5	2	4	3	1	1
2	1	0	4	9	1915	9	2006	0
1	0	0	0	0	0	0	0	0
0	0	0	0	0	0	0	0	0
712	583	4328	0	2	2	0	0	0
0	0	0	0	0	0	0	0	0
0	0	0	0	0	0	0	0	0
0	0	0	0	0	0	0	0	0
0	1	0	0	1	1	0	0	0
77939	41963	44971	46078	62778	63795	67158	59622	32495
48702	43853	30199	22959	47924	69320	55686	34861	37208
2750	2055	2292	1	0	5479	0	3	1359
39249	26654	54813	45474	65104	41305	21539	26670	14544

For Review Only

Nov20_N6	Nov20_N6	Nov20_N7	Nov20_N7	Nov20_N7	Nov20_N8	Nov20_N8	Nov20_N9	Nov20_N9
0	0	0	0	0	0	0	0	0
6057	19991	13156	3689	3570	6579	6960	12173	0
0	0	0	0	0	0	2	0	0
0	0	0	0	0	0	0	0	0
35168	20253	26798	3727	20363	66416	32	24	2
7	77	209	55	11	132	286	450	0
31	4242	19180	4	112	9988	4235	7530	0
0	0	0	0	0	0	0	0	0
20542	19494	12154	4544	15062	14402	48	1	0
0	0	0	0	0	0	0	0	0
9366	16981	7077	14078	7215	2664	7939	13056	5938
3411	21462	2446	610	1356	2	3	0	0
3506	7181	2008	41	655	11	0	0	0
305	1234	150	23	119	62	0	0	0
14403	14694	1464	2706	10792	6206	23	6	0
9	18	5861	0	0	0	0	0	0
0	0	0	0	0	0	0	0	0
5985	12413	9037	2710	9427	5257	9	6	0
0	0	0	0	0	0	0	0	0
2372	19	13	812	15	8	1	0	0
0	0	0	0	0	0	0	0	0
1	0	0	0	0	0	0	0	0
13026	5744	14388	806	15103	6863	3964	6943	12939
0	0	0	0	0	0	0	0	0
3	3	0	375	2000	0	1	0	0
0	0	0	0	0	0	0	4	0
6	14	3954	1799	7239	2642	2275	3853	0
1	6	2086	0	137	19	7276	12882	15306
0	0	0	0	0	1	0	0	0
0	0	0	0	0	0	0	0	0
0	0	0	1	17	2040	0	0	0
0	0	0	0	0	0	0	0	0
0	0	0	0	0	0	0	0	0
0	0	0	0	0	0	0	0	0
0	0	0	0	0	0	0	0	0
78399	63440	95425	18639	36856	22496	75	23	2
56951	76573	43180	16246	44797	24637	90	74	14915
8	1545	0	1811	3602	1967	8	0	0
46965	18398	15503	7571	21479	34984	59	32	0

For Review Only

Nov20_N9	Nov20_S5-	Nov20_S6-	Nov20_S6-	Nov20_S7-	Nov20_S1(	Nov20_S1:	Nov20_S1:	Nov20_S1:
1	0	1	1	0	0	0	0	0
11489	41088	16885	35400	238698	147155	149807	205166	131351
11	49496	0	24	1923	95	1	2	5
0	0	0	0	0	0	0	0	0
14	0	14	5160	0	3643	13	10775	11053
3497	1253	583	1470	13052	5693	5618	9232	5594
166	2628	151	10716	32675	12955	13285	29207	12463
1	5	26	246	21834	3	2	4	28
13	81160	2463	14774	13899	76492	33	38646	33393
0	0	0	0	0	0	0	0	0
34515	136163	26939	146842	13020	24558	65770	31449	73785
5	2	6	2440	3	22	5	18	56
2	0	0	5	2	3086	1	5	10
0	0	0	155	0	22	0	6	11
3	1	0	0	2	7	1	2	3
2	5021	2539	20682	19754	107	20093	6772	148
0	0	0	0	0	0	0	0	0
1	0	0	0	0	1	1	0	0
0	0	0	1	0	0	0	0	0
0	0	0	0	0	0	0	0	0
0	0	2	4	100	0	0	0	0
0	0	0	0	0	0	0	0	0
9	4845	2	0	0	21	3	68	30472
0	0	0	0	0	0	0	0	0
2	29987	2504	27925	74169	10350	12296	5223	11948
0	14773	3	13	3	6174	29	18	11
2	69330	11761	25336	19991	108250	96499	26575	10815
7	40991	5102	28196	7736	35711	4962	35169	26431
0	0	0	0	25	0	4	6	0
0	0	0	0	0	0	0	0	0
2	11	20	375	36026	124	25541	33711	4725
0	0	0	0	0	0	0	0	0
0	0	0	0	0	0	0	0	0
0	0	0	0	0	0	0	0	0
0	0	1	18	1783	3465	13408	28351	19702
22	0	0	0	0	9	5	5	5
7410	0	0	6	0	8	4	15	3228
1	888	12	225	18335	5785	5343	27	11172
15	6	17	4817	8390	49	5523	550	7515

For Review Only

Nov20_S13	Nov20_S14	Nov20_S14	Nov20_S15	Nov20_S15	Nov20_S15	Nov20_S16	Nov20_S16	Nov20_S16
0	0	2758	0	0	0	3	0	0
147598	12475	95710	40752	215415	214331	13467	11925	54333
0	4	2	1251	4569	0	1	8829	1
0	0	0	0	0	0	0	0	0
11468	8	10401	1689	9455	9183	5	11959	1
7304	446	3006	1810	12675	8867	621	28957	9661
53194	46	1676	2747	18657	9717	7698	8367	441
2	20437	72965	0	0	1	0	1	7
29857	2986	10947	12666	86285	53796	12314	9182	25
0	0	0	0	0	0	0	0	0
30703	22	10264	11174	52283	61574	58807	76357	254112
41	259066	10600	1	0	1	0	8	2
7	31110	10181	0	2	3	0	3081	0
5	124	268	1	11	8	0	24	0
3	3	0	1	5	2	0	0	0
10731	20961	38477	1438	5842	9887	10	10068	21
0	0	0	0	0	0	0	0	0
0	2	0	0	5	4953	50	0	1
0	0	0	1	2229	9	9402	0	0
0	0	0	0	0	0	0	0	0
0	0	0	0	0	0	0	0	0
0	0	0	0	0	0	0	0	0
2	3	0	1124	2	1	0	0	10
0	0	0	0	0	0	0	0	0
15	1408	38187	3592	4190	5	15	5	5
5	0	0	3	6	0	15	0	7
15462	15	6	3691	16534	10168	46122	1	15633
12	0	3	900	1	4	17	3335	29240
0	0	0	0	12	3	9	0	0
0	0	0	0	0	0	0	0	0
20	10	5	1783	50558	16188	33387	1	1
0	0	0	0	0	0	0	0	0
0	0	0	0	0	0	0	0	0
0	0	0	0	0	0	0	0	0
6444	1	6972	1231	0	1	0	0	7067
2	18	0	0	0	0	0	0	6
16	43	20	20	4	6	0	8	3
2204	11897	11477	2631	14	781	104932	1	11541
15	8949	6064	512	5807	9647	3	5373	10297

For Review Only

Nov20_S1	Nov20_S1	Nov20_S1	Nov20_S1	Nov20_S1	Nov20_S1	Nov20_S1	Jun21_M1	Jun21_M1
0	2	3	0	4	1082	1	0	0
31532	56643	156359	204526	26577	6022	596	2524	196
1	20	18316	9104	1953	0	58	0	0
0	0	0	0	0	0	0	0	0
7	17	7	4	7	18	18	2	9
1541	2986	5103	6351	1263	314	72	0	0
11110	4920	7218	1340	7622	1586	59	5	40
10	8872	76765	62753	1	2	35	0	0
18012	44748	17746	78778	234339	112840	721440	3	6
0	0	0	0	0	0	0	0	2
1	6	44	10740	13070	1866	602	2812	13
1	12	8	1	1	1	4	2769	14934
1	3	1	0	1	7	0	59	4616
0	4	0	0	0	3	0	2	148
1	3	8	11	8	11	44	0	0
9924	9576	68327	72772	4681	2264	157	2	0
0	0	0	0	0	0	0	0	2
0	0	6	3	5	6	39	0	0
0	0	0	0	0	0	0	0	0
0	0	0	0	0	0	0	0	0
0	0	0	0	0	0	0	0	0
0	0	0	0	0	0	0	0	0
7648	1	1	1	5	15	46	2278	1368
0	0	0	0	0	0	0	0	0
5467	9122	7	3227	11911	3066	211	3	26
16	19	0	0	9	2330	67	3	0
30139	44291	6	46	25279	10284	1012	2933	1
5	5377	3780	0	0	4769	3	2128	3730
0	0	0	0	4	2	1	0	0
0	0	0	0	0	0	0	0	0
3	1	7	28	17041	2093	155	1	0
0	0	0	0	0	0	0	0	0
0	0	0	0	0	0	0	0	0
0	0	0	0	0	0	0	0	0
1	0	0	2	1269	0	0	0	0
1	1	13	10	9	24	14	2	9
9	8537	19	13	16	1422	6	0	0
1	2	47	17649	8008	1	179	0	0
4	1561	5122	29418	466	1703	170	1226	19

For Review Only

Jun21_M1	Jun21_M2	Jun21_M2	Jun21_M2	Jun21_M3	Jun21_M3	Jun21_M3	Jun21_M4	Jun21_M4
0	0	0	0	0	0	0	0	0
26191	379409	6208	135788	243196	347755	139963	14611	58019
0	0	0	0	0	0	0	0	0
0	0	0	0	0	0	0	0	0
2457	0	1	0	1	44	12332	0	0
36	160	35	127	84	45	38	374	86
5938	16710	4025	17862	6009	667	10930	48036	11557
0	0	0	0	0	0	0	0	0
1959	7	71	14132	16814	8827	4220	44	11376
0	0	0	0	0	0	0	0	0
185	42497	135	39422	269	22220	14	2814	35
9596	4904	29	7529	1	16	2134	9	96
379	21833	29	6745	4	4	1209	6613	54
5	3	0	3	0	0	1	0	0
0	0	0	0	0	0	0	344	0
0	0	0	0	1	0	1	1	0
1	1	0	1	5	70	18094	0	0
0	0	0	0	68	0	0	0	0
0	0	0	0	0	0	0	0	0
0	0	1	19	4648	0	0	0	0
0	0	0	0	0	0	0	0	0
0	0	0	0	0	2	0	0	0
36	10446	7	1338	1227	12013	1	1	35
0	0	0	0	0	0	0	0	0
5939	445	14867	1	2	5	54	12653	131
0	0	229	5	0	0	0	0	1
11	140	18400	21074	2	0	5	0	27
101	28511	5743	28703	235	20002	1759	43	15013
0	0	0	0	0	0	0	0	0
0	0	0	0	0	0	0	0	0
2	0	0	1	0	0	0	0	1
0	0	0	0	0	0	0	0	0
0	0	0	0	0	0	0	0	0
0	2	0	0	179	0	0	1	0
2	0	1	0	1	0	0	0	3
2020	165	19360	10	15	320	82008	56	72
0	0	0	0	0	0	0	0	0
6	1	4	40	10545	1	10	4	90
5614	47	6084	6184	6	63	8102	7010	37397

For Review Only

Jun21_M4	Jun21_M5	Jun21_M5	Jun21_M5	Jun21_M6	Jun21_M6	Jun21_M6	Jun21_M7	Jun21_M7
0	0	0	0	0	0	0	0	0
97277	227162	111566	66057	385050	430828	279862	41281	17795
0	0	0	0	0	79	2	0	0
0	0	0	0	0	0	0	0	0
0	1	0	10	8481	206	18	18495	0
131	36	160	66	124	308	237	653	1
11932	775	13608	9386	10670	9443	20415	81080	35
0	0	0	0	0	0	0	0	0
5	37284	18628	3	1	17601	41	4996	27
0	0	0	0	0	13	0	0	0
3165	11557	14125	7	8505	13226	26991	3197	13474
15209	26037	60	85219	1	125	32	4813	1
7491	515	9	19918	0	53	12452	75	0
6	8	0	35	0	0	1	1	0
0	0	0	0	0	8	15	9573	0
0	0	0	0	1	946	7	5	2
0	4	0	0	0	3	0	0	0
0	0	0	0	0	174	0	1	4
0	0	0	0	0	9	0	0	0
0	0	0	0	0	43	0	0	0
0	0	0	0	0	0	0	0	0
0	0	0	0	0	0	0	0	0
5139	25511	10	22571	471	112	1	1829	30277
0	0	0	0	0	0	2	1225	0
15528	1	2	3	1	3660	23	10	15
0	0	0	0	0	54	0	0	0
0	1	0	0	0	2093	17	2976	9
2	11887	0	16	4227	1826	16	6	13
0	0	0	44	0	23	0	0	0
0	0	0	1	0	3	0	0	0
0	1	19	19914	0	6086	10	11	6
0	0	0	0	0	4	0	0	0
0	0	0	0	0	0	0	0	0
0	0	0	1	0	131	0	0	0
0	0	0	1	0	321	6	5043	1
6674	15	19392	3	1	209	1	1	2
0	0	0	0	0	0	0	0	0
11284	0	1	0	0	8302	9	5143	2
6492	17241	9	6	3141	7661	26	20461	1

For Review Only

Jun21_M7	Jun21_M8	Jun21_M8	Jun21_M8	Jun21_M9	Jun21_M9	Jun21_M9	Jun21_M1	Jun21_M1
0	0	0	0	0	0	0	0	0
138031	12168	120519	523283	178709	220855	1706	559642	655333
0	0	0	0	2	0	0	0	0
0	0	0	0	0	0	0	0	0
2	2	0	0	1	39	13191	0	6
35	0	247	94	121	669	1314	83	75
4198	74	15468	8260	9239	76948	10219	951	1152
0	3	0	0	0	0	0	0	0
46	31	46	56	43	4239	35	29	53
2	674	0	0	0	168	0	0	0
8762	2	14	4082	2914	2	4	14	4319
0	2	4	2190	1	0	1	0	2
1	0	0	5	1	0	0	0	0
0	0	0	1	0	0	0	0	0
0	0	0	1	0	0	1	0	0
8	7	10	5	4	4	2	4	3
0	0	0	10	5236	0	83	0	0
3	1	2	0	2	61	20819	2	0
0	0	1	1	1	0	0	0	0
0	1	1	0	2	32	9717	0	0
0	0	0	0	0	0	0	0	0
0	0	0	0	0	0	0	0	0
0	0	1	2	1	1	1	0	2
0	0	0	0	0	0	0	0	1
23	10	17	27	17	17	15	18	42
0	0	0	0	0	0	0	0	0
21	11	11	20	2360	12	12	6	12
9	8	14	14	12	14	5	7	4
0	0	0	0	0	0	0	0	0
0	0	0	0	0	0	0	0	0
18	9	10	20	6	6	9	14	10
0	0	0	193	0	315	0	0	0
0	0	0	0	0	0	0	0	0
3	0	20	6288	29	8745	1	4	5
2	0	4	3	4	0	2	2	0
1	3	2	21	11376	20333	27471	29	8876
0	0	0	0	0	0	0	0	0
10	3	5	4	213	3	2	4	3
3	60	12	2270	19273	15735	28083	660	1

For Review Only

**Jun21_M1**	**Jun21_M1**	**Jun21_M1**	**Jun21_M1**	**Jun21_M1**	**Jun21_M1**	**Jun21_M1**	**Jun21_N1-**	**Jun21_N1-**
0	0	0	0	0	0	0	0	0
569235	462312	466306	189984	566503	29770	216765	22856	20248
1	0	0	0	0	0	0	1	0
0	0	0	0	0	0	0	0	0
1	0	0	2	2	1	1	0	0
60	47	56	22	81	11	38	77	3809
1791	875	858	294	1400	47	404	12834	108021
0	0	0	0	0	0	0	0	0
55	3613	5550	74	6796	34	27	28	3158
0	0	0	0	37	12840	0	4340	0
4	0	2	10	3	84	17324	11696	3772
1	0	0	0	3	28	4627	11774	110
0	0	0	1	1	0	73	6901	27
0	0	0	0	0	0	2	62	0
0	0	0	0	0	0	0	2	191
4	4	1	46	4531	5	4	10	903
0	0	0	0	0	0	0	6	2047
1	2	1	0	1	1	1	0	0
0	0	0	0	0	0	0	0	0
1	0	0	1	0	0	0	0	0
0	0	0	0	0	0	0	0	0
0	0	0	0	0	0	0	0	0
1	0	32	13923	2400	225	33415	0	0
0	0	0	0	0	0	0	0	0
4304	14	16	21	29	20	14	8863	3634
0	0	0	0	0	0	0	0	3
9	6	8	8	9	10	10	6	1709
42	5682	8	1854	9620	11	3	0	1
0	0	0	0	0	0	0	0	2
0	0	0	0	0	0	0	0	0
15	11	7	17	7	9	10	4	25
1	205	0	0	0	0	8	0	0
0	2	0	0	0	0	0	0	0
34	7583	1	0	2	7	536	1	1
1	0	0	0	1	0	1	0	0
13	2741	2972	0	0	1	1	26	2806
0	0	0	0	0	0	0	0	0
4	4	3	44	6280	9891	10971	68	16014
0	3	2	4	119	29550	2	17	5949

For Review Only

Jun21_N1·	Jun21_N2·	Jun21_N2·	Jun21_N2·	Jun21_N3·	Jun21_N3·	Jun21_N3·	Jun21_N4·	Jun21_N4·
0	0	0	0	0	0	0	0	0
190	15	18	236	33218	33668	82374	7949	122
0	0	0	0	0	0	0	2	0
0	0	0	0	0	0	0	0	0
0	0	0	0	1	0	0	3	7
68	33	0	3	440	7	23	2	0
12466	3737	18	567	59431	46	190	19	2
0	0	0	0	0	0	0	0	0
46997	25	269	42469	89	13155	0	0	2
0	0	0	0	0	0	0	0	0
4	14	124	24114	28624	459	90913	9690	11124
14593	5	3	122	18817	23505	26	4901	0
2904	3	11	253	48331	734	1	122	1
8	0	0	0	15	17	0	2	0
5	441	0	0	0	0	0	1	1
22709	3	0	7	100	14681	1	1	3
162	24339	80	17832	1	4	536	0	0
0	0	0	0	0	0	0	0	0
0	1	0	0	0	0	0	0	0
0	0	0	0	0	0	1	0	0
0	0	0	0	0	0	0	0	0
1	0	0	0	0	0	0	0	0
0	0	0	0	1	1	1	0	1
0	0	0	0	0	0	0	0	0
26874	1	0	8	2	6	44	6727	1
0	2	0	1	2	51	11137	0	0
0	1	0	3	0	1	0	0	2
1	2	0	2	65	10574	15	1124	23279
2	0	0	0	25	0	0	0	0
0	0	0	0	3	0	0	0	0
4205	0	5	92	18878	0	1	2	0
0	0	4	430	0	4	496	3	369
0	0	0	1	0	0	1	0	7
2	7	148	25708	11	217	44184	316	41333
0	0	3	1	0	0	0	1	3
634	100200	23236	25008	126	17551	80696	13674	530
0	0	0	0	0	0	0	0	0
0	1	0	0	2	1	2	11	104
6	0	1	13	3	9	56	10872	1

For Review Only

Jun21_N4·	Jun21_N5·	Jun21_N5·	Jun21_N5·	Jun21_N6·	Jun21_N6·	Jun21_N7·	Jun21_N7·	Jun21_N7·
0	0	0	0	0	0	0	0	0
18136	22120	14674	97	10155	7650	277	2472	5449
0	1	2	0	0	0	2	0	0
0	0	0	0	0	0	0	0	0
113	24251	5853	54	7304	27	4390	17	1706
2	6	71	0	65	5	91	4	16
44	235	13144	50	7339	103	14710	2761	1996
0	53	6832	16	2348	0	0	10	1232
18	254	37404	16416	6863	2	4	8	75
5	23	4288	2	0	12	1638	7	240
20890	36285	8872	34302	20518	22	2795	41	4665
5	79	10310	2	23	3486	911	9	1357
1	1	324	1	1	69	1537	0	18
0	2	382	0	0	2	0	0	0
0	41	5239	0	0	0	0	0	0
3	0	1	0	0	2	14	6024	1199
4	58	9612	233	30344	4838	1	3	24
0	0	0	32	4513	0	10	0	0
0	0	0	0	0	0	0	0	0
1	1	5	34	4328	10	1745	0	3
0	0	0	0	0	0	0	0	0
0	0	0	0	0	0	0	0	0
21	5946	90	25008	0	0	0	2	1
0	0	0	0	0	0	0	0	0
3	45	5760	5	3	25	2313	5051	998
0	1	2	8	0	0	0	8	0
2	64	8019	21723	660	3	16	5559	2
45	7290	146	34639	7426	9	48	13	1135
0	0	0	0	0	18	1966	16	2
0	0	0	0	0	0	1	0	0
2	1	1	2	1	23	2586	7588	2783
0	118	0	2	60	0	0	0	0
0	3	0	0	0	0	0	0	0
56	12471	1	37	5231	0	1	4	24
34	6459	2	35	4458	0	0	0	0
95	13014	185005	1559	211861	18174	8679	3180	20383
0	0	0	0	0	0	0	0	0
19219	61	8445	28	2788	9	2369	1401	1274
20	387	55738	399	55970	3760	4126	5283	18292

For Review Only

Jun21_N8·	Jun21_N8·	Jun21_N8·	Jun21_N9·	Jun21_N9·	Jun21_N9·	Jun21_N1(	Jun21_N1(	Jun21_N1(
0	0	0	0	0	0	0	0	0
118386	85750	40138	105514	332453	321654	7678	316	13434
0	0	4	1	6	2	0	4	1
0	0	0	0	0	0	0	0	0
6046	19983	2934	5	1	2	1	1	5
197	185	180	174	582	431	5	11	23
25582	25876	23430	33968	83911	35557	617	2201	1520
0	15	2175	0	0	0	0	0	0
20245	11962	7680	7	22	1238	1391	1	0
0	146	9386	0	1	1	0	0	0
14528	14174	5021	185	21851	35953	636	1	1
7	2792	3688	8	81	12	3	1	1
1	23	1332	221	29757	3	685	0	1
0	0	64	0	0	0	0	0	0
0	0	2	0	0	0	0	0	0
0	0	0	0	0	1	1	0	0
103	24097	13398	0	3	1	1	0	0
0	135	1791	2	0	0	0	0	0
0	0	0	0	0	0	0	0	0
30	5726	2992	0	0	0	0	0	0
0	0	0	0	0	0	0	0	0
0	0	0	0	0	0	0	0	0
0	14	93	9411	30405	17435	1652	0	2
0	0	0	0	0	0	0	0	0
4250	4492	1296	10894	17267	4859	1140	2	0
0	0	0	0	0	0	0	0	0
6	2687	1468	6	3	1	1	0	1
11	2041	41	6073	120	9807	0	2	32
0	0	8	176	0	0	0	0	5
0	1	23	4872	1	0	0	0	0
1	3	104	17525	2	1	1	15	2350
109	90	0	0	0	0	0	0	0
0	0	0	0	0	0	0	0	0
5413	5048	25	5	3	4	44	0	0
0	17	2653	2	106	10312	0	0	0
4455	120824	106796	93	15	18	77	5558	1458
0	0	0	0	0	0	0	0	0
31	7348	1391	132	14876	8387	2	4	48
1959	89294	126971	10272	92	30	1391	9100	1138

For Review Only

Jun21_N1	Jun21_N1	Jun21_N1	Jun21_N1	Jun21_N1	Jun21_N1	Jun21_S1-	Jun21_S1-	Jun21_S1-
0	0	0	0	0	0	0	0	0
435931	475513	247627	576028	301483	389306	57019	108766	2695
0	0	4	1	3	3	0	0	0
0	0	0	0	0	0	0	0	0
669	0	7	24	2777	0	169	30720	1
233	307	449	50	367	2660	299	82	31
28116	34895	27384	1339	28026	21176	9304	9936	610
0	0	3	0	25	1738	0	0	0
126	46	7415	15	2035	12377	19535	154139	1444
0	0	0	0	1	0	0	0	1
0	1	12	4	4	46	9	164	16434
8	1	11	3	6	3	58	8062	36282
1	0	3	2	1	1	11	1828	368
0	0	0	0	0	0	0	18	4
0	0	0	0	0	0	0	0	0
1	3	3	0	0	0	8289	30564	18
1	16	2707	21	3870	1	0	0	0
0	2	3	3	1	0	706	110684	1
0	0	0	0	0	0	34	6148	20
1	0	0	0	0	0	118	17471	0
0	0	0	0	0	0	0	0	0
0	0	0	14	1796	0	0	0	0
2	1	41	3247	3156	0	2	0	1
0	0	0	0	0	0	0	0	0
1	32	5419	11	63	6595	572	89974	502
0	0	0	0	0	2	0	63	1
0	1	1	5	43	3550	209	34633	539
5083	54	7537	1	0	0	184	27708	10
0	0	0	0	0	0	0	12	0
0	0	0	0	0	0	0	3	0
1	0	2	2	4	1	47	6858	260
0	0	2	197	53	0	0	8	5
0	0	0	0	0	0	0	0	0
3	0	64	4638	2286	4	11	408	16857
0	1	2	1	24	1961	3	25	466
45021	114	15983	7819	33420	31	2	4	1
0	0	0	0	0	0	0	0	0
6981	1	3	1	1	0	6	11	128
15414	86	13422	2560	5779	182	10633	16	3

For Review Only

Jun21_S2-	Jun21_S2-	Jun21_S2-	Jun21_S3-	Jun21_S3-	Jun21_S3-	Jun21_S4-	Jun21_S4-	Jun21_S4-
0	0	0	0	0	0	0	0	0
219420	157082	157280	123719	79870	102669	200817	184270	185037
1	0	3	0	0	0	19	3157	2675
0	0	0	0	0	0	0	0	0
2	5	3	0	1	1	1	1	2
2227	3010	2515	3982	21	107	169	89	28
48503	64009	47064	724	231	10167	13346	10277	467
0	0	0	0	0	0	0	0	0
114004	175220	166305	117	11449	20398	3022	6764	13273
0	0	0	1	0	0	0	1	0
16034	8788	4928	5	9	163	32104	19719	5221
1	3	0	1	11	1	0	0	3
1	0	0	2	0	0	0	2	4
0	0	0	0	0	0	0	0	0
0	3	0	0	0	1	0	0	0
1275	48	4824	4514	34545	15101	157338	123809	153047
0	0	0	1	0	0	0	0	0
71	4	2	1	1	1	0	4	0
1342	0	0	0	0	0	0	1	0
1	0	0	0	0	0	0	0	1
0	0	0	0	0	0	518	0	0
0	0	0	0	0	0	0	0	0
0	0	0	44	6461	0	3	31	4068
0	0	0	0	0	0	0	0	0
39097	56589	94451	178523	142325	57410	127765	160701	180469
60	15	24	2	23	14	65	7	76
38661	24201	36162	2465	16206	5762	33487	20908	16064
7	11	14	15	26	263	45518	77781	36196
51	16	30	858	2816	724	38	59	42
6	0	0	69	45	53	0	7	4
18852	7546	11378	236829	336933	315151	18246	23074	21688
0	149	61	0	0	0	0	0	2
0	0	0	0	0	0	0	0	0
50	5550	2355	0	0	2	1	5	3
38218	16654	29117	2	2	24	4084	33	6346
1	1	4	0	5	4	1	0	2
0	0	0	0	0	0	0	0	0
11188	3213	15522	4	11	217	33754	27375	34147
32	2986	15	5554	1	2	1	2	2

For Review Only

**Jun21_S5-**	**Jun21_S5-**	**Jun21_S5-**	**Jun21_S6-**	**Jun21_S6-**	**Jun21_S6-**	**Jun21_S7-**	**Jun21_S7-**	**Jun21_S7-**
0	0	0	0	0	0	0	0	0
65415	25650	78959	119350	157740	131654	83333	116251	114529
4035	4224	38	6186	3078	0	1	1	14
0	0	0	0	0	0	0	0	0
2	3	2	1	0	2	1	2	5
7	18	209	19257	37730	313	5667	3295	28751
141	143	10347	3627	10038	23427	14293	13328	20512
0	0	0	0	0	0	0	0	0
143374	162841	146049	21549	17280	29774	218747	183557	186816
1	0	0	0	0	1	0	0	0
9	178	24820	3	0	1	4	6	52
1	4	13	3249	37	4820	0	2	3
0	2	6	584	0	74	1	0	0
0	0	0	3	0	2	0	0	0
1	1	0	1	0	0	3	0	0
35	10798	8971	8156	21227	17510	17854	17054	7762
0	0	1	0	0	0	0	2	0
0	3	4	2	0	3	1	0	1
0	0	0	0	0	0	0	0	0
0	1	0	1	0	1	0	0	0
0	0	0	0	0	0	0	0	0
0	0	0	0	0	0	1	1	0
50	14678	2	1	2	2	0	0	10
0	0	0	0	0	0	0	0	0
225681	214033	136274	185882	81154	136430	173328	156995	150458
22	21	45	103	4	25	5342	4859	6079
24035	15760	32111	50061	7314	2358	118486	126100	102789
53292	41215	29599	165745	128338	156978	61487	43150	58632
161	159	39	30	20	13	39	40	49
7	5	2	0	1	0	4	1	1
35655	58438	33173	8615	8576	3348	12934	12657	25030
0	0	1	0	0	1	1	0	0
0	0	0	0	0	0	0	0	0
1	0	0	0	2	1	4	2	3
2	1	1	0	1	2	4	1	4
0	2	3	39	3415	7391	3	615	2
0	0	0	0	0	0	0	0	0
47	9664	27	5482	52	12552	3618	11458	8843
3	19	8	49	6483	1492	10	12	10

For Review Only

Jun21_S8-	Jun21_S8-	Jun21_S8-	Jun21_S9-	Jun21_S9-	Jun21_S9-	Jun21_S10	Jun21_S10	Jun21_S10
0	0	0	0	0	0	0	0	0
16506	18801	9968	14578	35092	26916	11681	34296	30860
1954	2331	0	0	0	1	1	1	0
0	0	0	0	0	0	0	0	0
16	1726	23	1814	2	4	1	1	1
44	108	5610	73	1805	1028	3	6	189
6904	12519	4326	7365	7262	7120	41	203	20046
0	0	0	0	0	0	0	0	0
122151	86335	73195	383304	341203	477960	5246	43	121
0	1	1	6	1001	0	2	0	0
8444	2	19	1862	2	2	2	3	2
4	1	5	770	0	1	3	162	20598
0	0	2	27	1	1	1	11	887
0	2	0	6	0	0	0	0	1
0	0	0	0	2	0	0	0	0
14037	7589	6261	7	6	11	19	51	1013
0	0	0	0	0	1	2	10	2181
2	1	17	1372	3	2	2	7	231
0	0	0	0	0	0	0	0	0
0	0	0	18	2	0	4	7	72
0	0	0	0	0	0	0	0	0
0	0	0	1	0	1	0	0	0
0	0	1	1	1	1	0	0	0
0	0	0	0	0	0	0	0	0
100009	98032	53853	64243	36039	39932	27	78	1170
6446	1463	2743	207	567	4543	0	3	0
153429	128020	375060	38740	26287	40858	11	11	69
179704	125560	97617	84078	52699	65409	9	15	235
253	184	95	24	17	25	1	29	1
10	8	10	3	0	2	0	1	1
92033	63493	39187	9369	4633	9847	193	23351	140
0	0	0	1	36	0	0	0	0
0	0	0	0	0	0	0	0	0
0	2	2	13	2100	1	0	3	0
1	3	27	2123	1482	1	0	1	1
1	3	8	958	1504	669	13561	21516	2
0	0	0	0	0	0	0	0	0
2633	1671	4058	4336	3985	1531	13	28	355
11	4	14	664	916	43	3451	234	27151

For Review Only

Jun21_S11	Jun21_S11	Jun21_S11	Jun21_S12	Jun21_S12	Jun21_S12	Jun21_S13	Jun21_S13	Jun21_S13
0	0	0	0	0	0	0	0	0
262416	225103	192462	178686	162119	140818	257298	351887	229010
72	10498	0	8	1289	0	1	0	4
0	0	0	0	0	0	0	0	0
5	14	123	13652	17795	36953	35674	302	32168
7507	545	107	548	346	2621	9689	489	633
39503	61805	932	75993	50120	93678	72749	66164	88307
0	0	0	0	0	0	1	2	1
7345	13344	11661	36632	51345	31723	49398	37754	11083
0	0	0	0	0	0	1	0	0
3	3	33	39	4514	5036	46765	190	17723
29	897	26	1266	17624	2675	31	4753	2
7152	17	38	4340	2724	3608	60	11318	1
0	0	0	1	6	5	2	1	0
0	0	0	0	0	1	0	0	1
106560	107973	6885	18	1646	88	10630	11884	544
0	0	0	0	0	0	0	0	0
24777	13163	33578	480	23	2907	3	3	2
0	0	29	4387	0	0	0	0	0
10084	8639	12005	3	0	59	0	1	4
0	0	0	0	0	0	0	0	0
0	0	0	0	0	0	1	0	0
0	0	4	3	34	5046	1	0	1
0	0	0	0	0	0	0	0	0
131540	82106	131957	126829	63174	108578	9673	6356	621
11	13	49	123	100	81	3	4	3
5036	22667	34161	121155	64760	53980	122	18227	350
24145	12477	114	2192	6353	4400	7885	15051	254
30	110	84	360	200	213	120	37	267
1	5	10	1096	14	8	6	6	16
15236	55474	27118	113353	74516	89415	49155	21352	97548
1	50	1	0	0	0	0	5	308
0	0	0	0	0	0	0	0	0
22	3415	10	3	2	1	7	153	14643
3	13	149	21088	13399	11012	0	1	36
33	1	7	5	827	11	1543	5	3
0	0	0	0	0	0	0	0	0
35383	46317	29922	2612	4804	13319	14344	5427	6972
2082	3	7	4	6	5	89	12996	2124

For Review Only

Jun21_S14	Jun21_S14	Jun21_S14	Jun21_S15	Jun21_S15	Jun21_S15	Jun21_S16	Jun21_S16	Jun21_S16
0	0	0	0	0	0	0	0	0
289167	238376	168594	126672	182405	171151	141756	50450	274000
96	2972	497	0	12	568	4	80	7914
0	0	0	0	0	0	0	0	0
54	2746	3694	1	4	1	2	0	3
100	3113	860	1292	1132	536	114	306	27750
3067	12693	7545	4829	11253	8599	9987	1898	115980
2	4	0	0	0	0	0	0	0
188465	81816	96133	227803	326857	207058	100793	240463	83219
0	0	0	0	0	0	0	0	0
2146	37	9	1208	1153	4	3	3	2
2	13	2	3	1	1	5	52	4253
3	3	1	1	0	0	1	0	89
0	1	0	0	0	0	1	0	1
2	1	0	0	1	0	0	0	0
62398	46499	29487	960	683	1125	9	5	4
0	0	0	0	0	0	0	0	0
56	1	10	0	2	0	2	40	3563
0	0	1	0	0	0	0	0	0
14	1647	1146	0	0	0	0	0	61
0	0	0	0	0	0	0	0	0
2	0	0	0	0	0	0	1	2
4	2	2	0	8	711	6534	0	1
0	0	0	0	0	0	0	0	0
66826	57810	80639	942	63	3992	24	65	3879
15	3	10	77	3669	1040	58	73	4062
13456	5335	14873	56785	62926	75040	11472	21184	29446
12811	11938	6241	52772	37258	43541	56773	12135	47910
8	16	22	9	10	18	0	0	43
1	1	1	0	1	3	0	0	2
5384	3824	7643	4840	6294	5148	12	119	13196
79	878	0	0	37	33	0	3	0
0	14	0	0	0	0	0	0	0
3238	57079	18	33	3087	1112	1	0	0
60	6680	39	5165	6856	8730	0	4	2
3	1	2	1	1	1	0	1	1
0	0	0	0	0	0	0	0	0
2830	14704	3670	46312	28548	33015	22687	244	22753
39	21	28	1940	13	10	15	345	7

For Review Only

Jun21_S17	Jun21_S17	Jun21_S17	Jun21_S18	Jun21_S18	Jun21_S18	Jun21_S19	Jun21_S19	Jun21_S19
0	0	0	0	0	0	0	0	0
173003	116419	100854	15293	34948	1062	104272	84580	115688
4607	1320	9310	1	1	3	24	11471	6223
0	0	0	0	0	0	0	0	0
249	3	3	0	2	19	2308	2	0
9767	10095	3294	0	51	5	158	1014	2216
26817	15082	17358	202	10293	167	16680	14216	19526
0	0	0	0	0	0	0	0	2
167163	395508	463812	164	24568	736	90148	144536	111947
15	0	0	0	0	0	0	0	22
2548	3672	1	2	44	10373	6	2755	18
164	4	3	101	11966	19372	9	4215	12
46	1	0	12	1330	1721	0	67	1
0	0	0	0	1	8	0	1	0
16	0	0	0	0	0	0	0	2
1162	1928	1951	4	2	29	3449	4	1652
10	0	0	0	0	0	0	0	2191
208	3	0	1	0	0	0	1	16
17	0	0	0	0	0	0	0	0
68	3	0	0	0	0	0	0	5
3	0	0	0	0	0	0	0	0
1	2	1	0	0	0	0	0	0
124	2	684	0	0	1	0	1	1472
1	0	0	0	0	0	1	0	0
22825	16884	11983	17	22	320	36283	85320	25941
2228	18	23	0	0	1	12	3083	21
33059	17284	11523	14	23	139	22358	30711	13600
79827	31399	29565	17	30	261	27051	51230	26416
60	80	65	1	0	0	60	91	18
2	4	2	0	0	0	5	6	0
24910	31763	22880	12	29	190	21292	21102	6751
21	3	0	0	1	1	120	41	87
0	0	0	0	0	0	3	0	2
703	751	4	0	10	50	6297	2470	4818
3398	2698	1832	0	4	2	3	1	3
262	14	52	25838	41210	19452	1	0	176
0	0	0	0	0	0	0	0	0
3682	5145	2231	4	9	45	5133	8401	20182
872	1460	2952	9310	1	8	804	2733	127

For Review Only

Jun21_S20	Jun21_S20	Jun21_S20	Sep21_M3	Sep21_M4	Sep21_M5	Sep21_M5	Sep21_M1	Sep21_M1
0	0	0	0	0	0	0	0	0
8451	284016	345588	2602	23109	15797	2900	3624	109234
0	16	4622	125	141	147	132	127	106
0	0	0	3	4	21	3006	2	5
0	15	0	11851	41	52	33	34	41
97	2920	3021	54	166	85	73	55	212
13532	19873	1395	1365	18384	727	829	494	1289
16	3351	0	0	0	0	0	0	0
142	18999	54398	2138	2200	2281	2373	1882	2859
7316	0	0	30	34	32	48	30	42
6813	2	8	46491	37885	191154	8382	2759	69196
18	31	11681	376	7488	306	364	275	614
1	6	1390	25	42	28	32	37	55
0	0	26	12	9	5	5	4	11
0	0	0	0	0	0	0	0	0
22	3307	11770	840	830	1037	988	701	688
0	0	0	0	0	0	0	0	0
5837	0	0	42	25	34	25	31	17
0	0	0	0	0	0	0	0	0
2058	0	0	14	8	5	14	7	6
0	0	0	0	0	0	0	0	0
0	0	0	0	0	0	0	0	0
0	1	11	188	7333	25515	108	84	65
0	0	0	0	0	0	0	0	0
329	57694	57635	842	791	950	943	692	670
5	1207	0	37	38	55	50	40	41
11709	25	16	1776	1724	2027	2199	1505	1605
25	3640	9	8787	15921	13575	3409	2199	2031
0	0	0	0	0	0	0	0	0
0	0	0	0	0	0	0	0	0
1	0	0	547	578	631	712	507	492
15	24	0	383	282	397	979	60559	1233
2	0	0	0	0	0	0	0	0
8381	620	0	0	0	0	0	0	0
0	1	0	2063	2161	2479	2358	1977	1877
67481	1666	1	0	0	0	0	0	0
0	0	0	0	0	0	0	0	0
44	7816	9721	458	1366	35151	4786	378	317
39956	1575	9	37619	3874	5349	4879	4536	137811

For Review Only

**Sep21_N1-**	**Sep21_N2-**	**Sep21_N2-**	**Sep21_N4-**	**Sep21_N4-**	**Sep21_N5-**	**Sep21_N5-**	**Sep21_N5-**	**Sep21_N6-**
0	0	0	0	0	0	0	0	0
1871	22386	1773	28192	48975	26644	1639	2618	2363
74	165	96	89	77	88	104	105	78
0	4	1	3	1	4	2	4	2
41	45	21	28	62	32	28	43	32
32	77	35	536	160	4267	49	581	287
485	901	491	15638	693	926	695	25685	11209
0	0	0	0	0	0	0	0	0
189824	13396	1340	1515	2312	1669	1945	1988	1548
63	57	37	18	51	27	26	29	32
1122	16759	235947	96701	1998	61273	25372	63956	22416
70982	344	316	1341	105957	201	173	306	6317
7207	41	21	36	1216	29	25	21	195
658	8	4	38	2978	7	3	12	641
0	0	0	0	0	0	0	0	0
434	931	639	553	513	553	545	712	470
0	0	0	0	0	0	0	0	0
18	43	19	21	24	29	30	21	20
0	0	0	0	0	0	0	0	0
4	12	7	3	8	10	7	6	8
0	0	0	0	0	0	0	0	0
0	0	0	0	0	0	0	0	0
38	92	393	30452	48	62	42	54	44
0	0	0	0	0	0	0	0	0
543	1077	529	525	734	649	618	700	554
205	86	38	34	26	24	60	89	24
46338	26634	1193	1265	1197	1283	2362	13032	984
1525	3423	14564	12735	1720	1995	12565	8689	14731
0	0	0	0	0	0	0	0	0
0	0	0	0	0	0	0	0	0
341	763	351	385	431	405	361	463	323
269	495	338	1418	109042	268	191	248	223
0	0	0	0	0	0	0	0	0
0	0	0	0	0	0	0	0	0
4169	377057	1076	2180	1384	1927	1654	1749	1205
0	0	0	0	0	0	0	0	0
0	102	0	0	1	0	0	0	0
265	663	303	10972	330	524	15390	311	254
1826	5061	4091	2410	5385	375849	357121	379679	478722

For Review Only

**Sep21_N1**	**Sep21_S2-**	**Sep21_S2-**	**Sep21_S2-**	**Sep21_S3-**	**Sep21_S3-**	**Sep21_S4-**	**Sep21_S4-**	**Sep21_S5-**
0	0	0	0	0	0	0	0	0
3394	146077	138142	134450	30144	22392	51736	63143	107976
120	4170	410	7968	980	4699	4660	142	263
3	8	3	2	2	3	10	5	18
37	9755	189	4237	337	113	50	42	94
41	1074	5363	10957	193	155	177	228	459
946	23781	2548	10260	1852	1351	2901	2601	8485
0	0	0	0	0	0	0	0	0
3931	43390	95733	60572	6156	4936	8168	4462	17676
42	53	97	57	87	76	65	47	67
1819	9581	8031	10688	3601	4454	99052	122055	193145
2553	4625	454	334	585	512	298	266	401
47	101	8066	41	86	82	32	22	53
7	57	20	16	19	23	7	3	16
0	0	0	0	0	0	0	0	0
800	10171	13498	17660	93614	13701	97648	105636	52249
0	0	0	0	0	0	0	0	0
28	52	47	50	86	1916	33	95	13687
0	0	0	0	0	0	0	0	0
8	6	13	13	14	20	13	5	78
0	0	0	0	0	0	0	0	0
0	0	0	0	0	0	0	0	0
70	67	101	63	110	163	110	106	6107
0	0	0	0	0	0	0	0	0
695	14635	18731	12430	2382	8073	14305	9331	23450
46	185	273	320	104	1257	555	168	152
1546	43141	113765	122746	5415	28322	69394	37929	8896
2769	15040	26314	3244	6344	7079	196699	45997	63575
0	0	0	0	0	0	0	0	0
0	0	0	0	0	0	0	0	0
442	10558	15294	4221	22359	34423	5575	20417	1150
270	760	476	362	532	566	332	283	384
0	0	0	0	0	0	0	0	0
0	0	0	0	0	0	0	0	0
1972	148016	214540	162073	444652	395049	86296	93506	116704
0	0	0	0	0	0	0	0	0
0	0	1	0	0	0	0	0	0
2494	2553	24603	17343	7007	894	12202	437	8951
3016	11794	6720	18416	35430	4473	13686	5371	9877

For Review Only

Sep21_S5-	Sep21_S5-	Sep21_S6-	Sep21_S6-	Sep21_S7-	Sep21_S7-	Sep21_S7-	Sep21_S9-	Sep21_S10
0	0	0	0	0	0	0	0	0
71057	73875	22537	19754	7602	5546	6151	26637	93962
2713	4526	136	168	5682	9738	7092	145	233
4	2	2	4	2	8	2	3	0
56	44	54	55	68	72	151	21746	47
4436	2478	92	88	65	84	89	265	218
19695	10725	833	703	689	896	1411	9604	930
0	0	0	0	0	0	0	0	0
14410	4849	2534	7437	50858	78441	71101	370842	6241
58	41	40	35	46	48	40	77	35
252540	167048	34868	8620	1913	4180	1492	12049	51313
302	226	4998	228	377	357	290	400	304
35	28	147	22	27	32	35	77	25
9	4	9	6	9	10	8	14	17
0	0	0	0	0	0	0	0	0
24678	21317	22777	15246	11280	8065	7122	801	733
0	0	0	0	0	0	0	0	0
4523	1231	49	49	37	56	30	65	3089
0	0	0	0	0	0	0	0	0
25	12	7	11	7	7	13	13	20
0	0	0	0	0	0	0	0	0
0	0	0	0	0	0	0	0	0
77	55	207	107	62	125	651	66	66
0	0	0	0	0	0	0	0	0
22151	11575	6602	10122	11193	9784	11532	7116	97241
166	98	380	570	6710	3994	4961	3174	110
31969	12674	41091	66546	215366	211551	184638	80908	1919
63573	47783	297808	281914	240917	306343	244842	140359	117491
0	0	0	0	0	0	0	0	0
0	0	0	0	0	0	0	0	0
836	1887	18622	41914	1259	915	2840	5823	24646
342	412	298	303	385	308	306	421	334
0	0	0	0	0	0	0	0	0
0	0	0	0	0	0	0	0	0
117830	88288	70582	35462	13577	32658	43543	25408	60374
0	0	0	0	0	0	0	0	0
0	0	0	0	1	0	0	0	0
22097	5339	441	1794	361	589	525	435	326
7955	4895	2614	2405	2491	3070	5900	3800	55310

For Review Only

Sep21_S17	Sep21_S18	Sep21_S18	Sep21_S18	Sep21_S20	Sep21_S20	Sep21_S20-3
0	0	0	0	0	0	0
3627	91923	214272	172142	76082	76463	97964
41129	2330	202	2891	1000	5010	6282
2	5	2	2	1	2	4
100	107	113	72	198	76	1368
286	494	882	1173	1854	3884	4310
10897	33261	58609	77214	40067	26178	28046
0	0	0	0	0	0	0
378501	41803	4489	10945	117785	142553	138446
84	114	25397	46	77	78	86
1481	1785	2057	1464	1644	1263	1698
388	340	383	288	2222	2500	3411
42	38	43	62	4551	1342	158
10	10	18	14	57	82	48
0	0	0	0	0	0	0
1222	75897	125676	84794	25761	19917	18678
0	0	0	0	0	0	0
32	46	233	4569	42	612	42
0	0	0	0	0	0	0
9	27	7009	11	21	23	21
0	0	0	0	0	0	0
0	0	0	0	0	0	0
66	68	89	75	4714	1442	195
0	0	0	0	0	0	0
18050	59178	50268	23644	85433	76199	58891
50	167	110	2601	2252	2754	1095
9212	50912	25806	85173	62696	25291	36899
2546	7637	41288	13819	28965	15480	18742
0	0	0	0	0	0	0
0	0	0	0	0	0	0
725	27522	820	7035	76288	64222	56154
362	395	483	351	428	335	412
0	0	0	0	0	0	0
0	0	0	0	0	0	0
2518	2823	2782	9083	10320	6572	8215
0	0	0	0	0	0	0
0	0	0	0	0	0	0
429	4097	13962	17933	22090	8990	4147
2565	2903	10057	3177	13604	2100	2863

For Review Only

Table S5. Number of clean sequence reads of fish taxa in the negative controls.

**Taxa**	**FB-Jun21_M2**	**FB-Jun21_M12**	**FB-Jun21_N3**	**FB-Jun21_N12**
Carassius auratus	104797	25	10951	483
Channa argus	0	7	29	955
Clarias fuscus	0	0	0	0
Ctenopharyngodon idella	1	0	0	1
Cyprininae	18	0	57	95
Cyprinus carpio	117	31	8067	19619
Hemiculter leucisculus	2	7	9707	611
Homatula spp.	0	0	0	0
Hypomesus olidus	3	139	8127	1
Hypophthalmichthys spp.	30	1557	303	12
Hypophthalmichthys molitrix	0	23	0	2
Hypophthalmichthys nobilis	0	1	0	0
Micropercops swinhonis	0	1	0	0
Micropterus salmoides	1	0	0	0
Misgurnus spp.	0	0	0	0
Misgurnus dabryanus	0	0	0	0
Neosalanx taihuensis	22	0	0	0
Pseudorasbora parva	162	47496	1	3
Rhinogobius spp.	0	0	0	0
Rhinogobius cliffordpopei	0	0	2	0
Rhinogobius giurinus	1	1	1	0
Rhodeus sinensis	3	0	1	1
Schizothorax spp.	0	0	0	0
Schizothorax taliensis	1	1	0	0
Silurus spp.	0	0	0	0
Siniperca spp.	3	3	5302	2
Squaliobarbus curriculus	0	0	0	0
Tachysurus fulvidraco	0	1	0	1

For Review Only

**FB-Sep21_N1**	**FB-Sep21_N2**	**FB-Sep21_N4**	**FB-Sep21_N5**	**FB-Sep21_N6**	**FB-Sep21_S4**
43702	2769	48069	1541	2639	2147
99	141	118	92	124	132
0	1	4	3	3	2
32	38	55	28	40	35
81	61	820	44	69	54
626	938	30814	415	699	553
1549	1956	2017	1550	2026	1851
32	34	21	23	35	37
1312	2196	1595	1261	1796	1903
220	420	18162	185	254	220
13	28	40	20	23	28
9	6	49	6	8	9
650	991	7271	593	817	877
0	0	0	0	0	0
23	30	32	26	24	28
11	5	7	8	6	4
50	50	52	46	56	76
585	803	718	596	905	780
23	40	28	26	27	33
1313	1887	1652	1240	1753	1682
1906	3052	2574	1896	3217	2468
440	592	539	391	551	593
216	326	319	1018	122640	251
0	0	0	0	0	0
1520	2361	2040	1603	2460	2047
0	0	0	0	0	0
0	0	1	0	0	0
85050	497	9395	49658	447	383

For Review Only

Table S6. The fish taxa detected in shore, nearshore, and midline sites of the Erhai Lake in four sampling

Code[a]	Occurrence							
	Summer							
	Jun 2020				Jun 2021			
	Shore	Nearshore	Midline	Total	Shore	Nearshore	Midline	Total
CAR	10	0	0	10	12	0	0	12
OLA	0	0	0	0	0	0	1	1
MSA	2	1	4	7	2	8	2	12
SIN	0	1	0	1	7	11	8	26
HOM	0	0	0	0	2	4	2	8
MAN_MDA	16	5	2	23	7	2	1	10
MAN	9	0	0	9	3	0	0	3
MDA	14	7	1	22	4	3	2	9
ACH	0	0	0	0	0	0	0	0
CAU	20	12	12	44	20	12	12	44
CID	3	1	0	4	7	7	5	19
CAU_CCA	8	7	8	23	17	7	4	28
CCA	19	12	11	42	20	11	12	43
HLE	20	9	3	32	20	11	11	42
HMO_HNO	6	9	12	27	11	7	8	26
HMO	7	7	12	26	7	9	6	22
HNO	0	0	2	2	0	1	1	2
MAM	0	0	0	0	0	3	2	5
MPI	0	0	0	0	0	1	0	1
PPA	20	8	7	35	20	9	5	34
ROC_RSI	0	0	0	0	1	1	0	2
ROC	0	0	0	0	1	1	0	2
RSI	20	4	3	27	19	5	2	26
SLI_STA	0	0	0	0	1	3	0	4
STA	0	0	0	0	10	8	4	22
SCU	1	1	0	2	0	0	0	0
GAF	7	3	0	10	1	5	0	6
MSW	20	6	3	29	17	3	2	22
RCL_RGI	11	0	0	11	8	1	1	10
RCL	20	3	1	24	19	6	5	30
RGI	20	10	9	39	18	9	8	35
HOL	12	11	10	33	14	10	11	35
NTA	6	8	8	22	8	4	9	21
CFU	0	0	0	0	0	0	0	0
SIL	9	0	0	9	8	5	1	14
TFU	16	3	5	24	18	9	6	33
Taxa number	24	21	18	26	29	30	26	33

[a]The fish taxa corresponding to the codes are shown in Table 1.

For Review Only

events.

of fish taxa

Autumn							
Nov 2020				Sep 2021			
Shore	Nearshore	Midline	Total	Shore	Nearshore	Midline	Total
6	1	3	10	8	2	4	14
0	0	0	0	0	0	0	0
0	0	0	0	0	0	0	0
0	6	0	6	0	0	0	0
0	0	0	0	1	1	1	3
1	6	2	9	5	1	0	6
2	0	0	2	0	0	0	0
0	3	0	3	1	0	0	1
2	0	2	4	0	0	0	0
12	9	4	25	11	5	5	21
6	6	1	13	3	1	1	5
12	6	3	21	4	3	3	10
12	8	2	22	11	5	5	21
11	7	2	20	11	5	5	21
2	4	2	8	3	5	5	13
3	3	2	8	2	3	0	5
0	1	0	1	0	2	0	2
0	6	0	6	0	0	0	0
0	0	0	0	0	0	0	0
11	3	2	16	11	5	5	21
0	0	0	0	0	0	0	0
0	0	0	0	0	0	0	0
7	3	1	11	11	5	5	21
0	0	0	0	2	4	5	11
0	0	0	0	0	0	0	0
3	7	0	10	0	0	0	0
4	0	1	5	0	0	0	0
11	3	1	15	11	5	5	21
3	0	0	3	6	1	1	8
10	5	1	16	11	5	5	21
11	6	4	21	11	5	5	21
11	8	3	22	11	5	5	21
4	8	3	15	3	3	4	10
0	0	0	0	0	0	1	1
8	0	0	8	11	5	5	21
10	6	1	17	7	4	5	16
23	22	19	27	22	22	20	24

For Review Only

Table S7. α diversity index of 134 eDNA samples collected from different seasons and locations in four sampling events.

Sample name	Time	Season	Location	Richness index	Shannon index	Simpson index
Jun20_M1	Jun_20	Summer	Midline	9	1.68	0.78
Jun20_M10	Jun_20	Summer	Midline	6	1.28	0.68
Jun20_M11	Jun_20	Summer	Midline	12	2.02	0.82
Jun20_M12	Jun_20	Summer	Midline	10	2.03	0.85
Jun20_M2	Jun_20	Summer	Midline	12	2.14	0.86
Jun20_M3	Jun_20	Summer	Midline	8	1.72	0.79
Jun20_M4	Jun_20	Summer	Midline	14	2.17	0.84
Jun20_M5	Jun_20	Summer	Midline	8	1.39	0.71
Jun20_M6	Jun_20	Summer	Midline	12	2.06	0.84
Jun20_M7	Jun_20	Summer	Midline	6	0.47	0.2
Jun20_M8	Jun_20	Summer	Midline	5	0.79	0.39
Jun20_M9	Jun_20	Summer	Midline	11	2.02	0.84
Jun20_N1	Jun_20	Summer	Nearshore	8	1.29	0.65
Jun20_N10	Jun_20	Summer	Nearshore	12	1.9	0.8
Jun20_N11	Jun_20	Summer	Nearshore	8	1.17	0.53
Jun20_N12	Jun_20	Summer	Nearshore	13	2.07	0.83
Jun20_N2	Jun_20	Summer	Nearshore	7	1.34	0.69
Jun20_N3	Jun_20	Summer	Nearshore	11	1.93	0.82
Jun20_N4	Jun_20	Summer	Nearshore	10	1.99	0.84
Jun20_N5	Jun_20	Summer	Nearshore	9	1.51	0.68
Jun20_N6	Jun_20	Summer	Nearshore	11	0.94	0.36
Jun20_N7	Jun_20	Summer	Nearshore	15	2.41	0.89
Jun20_N8	Jun_20	Summer	Nearshore	14	2.23	0.86
Jun20_N9	Jun_20	Summer	Nearshore	10	1.98	0.83
Jun20_S1	Jun_20	Summer	Shore	17	2.26	0.85
Jun20_S10	Jun_20	Summer	Shore	13	1.61	0.73
Jun20_S11	Jun_20	Summer	Shore	16	1.92	0.79
Jun20_S12	Jun_20	Summer	Shore	17	1.71	0.72
Jun20_S13	Jun_20	Summer	Shore	16	1.79	0.77
Jun20_S14	Jun_20	Summer	Shore	20	1.75	0.71
Jun20_S15	Jun_20	Summer	Shore	14	2.06	0.83
Jun20_S16	Jun_20	Summer	Shore	15	1.67	0.71
Jun20_S17	Jun_20	Summer	Shore	15	1.72	0.72
Jun20_S18	Jun_20	Summer	Shore	12	1.69	0.77
Jun20_S19	Jun_20	Summer	Shore	15	1.79	0.77
Jun20_S2	Jun_20	Summer	Shore	18	2.05	0.81
Jun20_S20	Jun_20	Summer	Shore	16	2.03	0.85
Jun20_S3	Jun_20	Summer	Shore	11	1.35	0.62
Jun20_S4	Jun_20	Summer	Shore	15	1.58	0.73
Jun20_S5	Jun_20	Summer	Shore	13	1.53	0.67
Jun20_S6	Jun_20	Summer	Shore	11	1.86	0.81
Jun20_S7	Jun_20	Summer	Shore	13	0.88	0.35
Jun20_S8	Jun_20	Summer	Shore	14	1.87	0.78
Jun20_S9	Jun_20	Summer	Shore	15	1.56	0.64
Jun21_M1	Jun_21	Summer	Midline	13	2.05	0.82
Jun21_M2	Jun_21	Summer	Midline	12	1.83	0.76
Jun21_M3	Jun_21	Summer	Midline	13	1.1	0.44
Jun21_M4	Jun_21	Summer	Midline	13	1.69	0.73
Jun21_M5	Jun_21	Summer	Midline	10	1.62	0.68
Jun21_M6	Jun_21	Summer	Midline	13	0.62	0.23

For Review Only

Jun21_M7	Jun_21	Summer	Midline	13	1.58	0.71
Jun21_M8	Jun_21	Summer	Midline	8	0.33	0.14
Jun21_M9	Jun_21	Summer	Midline	13	1.56	0.69
Jun21_M10	Jun_21	Summer	Midline	5	0.08	0.02
Jun21_M11	Jun_21	Summer	Midline	7	0.26	0.09
Jun21_M12	Jun_21	Summer	Midline	11	0.93	0.41
Jun21_N1	Jun_21	Summer	Nearshore	16	2.15	0.84
Jun21_N2	Jun_21	Summer	Nearshore	10	1.18	0.55
Jun21_N3	Jun_21	Summer	Nearshore	15	2.23	0.87
Jun21_N4	Jun_21	Summer	Nearshore	11	1.96	0.84
Jun21_N5	Jun_21	Summer	Nearshore	19	2.33	0.87
Jun21_N6	Jun_21	Summer	Nearshore	17	1.49	0.62
Jun21_N7	Jun_21	Summer	Nearshore	19	2.41	0.88
Jun21_N8	Jun_21	Summer	Nearshore	19	1.93	0.78
Jun21_N9	Jun_21	Summer	Nearshore	13	1.36	0.57
Jun21_N10	Jun_21	Summer	Nearshore	13	1.61	0.72
Jun21_N11	Jun_21	Summer	Nearshore	10	0.63	0.27
Jun21_N12	Jun_21	Summer	Nearshore	14	0.57	0.21
Jun21_S1	Jun_21	Summer	Shore	19	2.25	0.86
Jun21_S2	Jun_21	Summer	Shore	13	1.83	0.79
Jun21_S3	Jun_21	Summer	Shore	10	1.31	0.65
Jun21_S4	Jun_21	Summer	Shore	13	1.87	0.8
Jun21_S5	Jun_21	Summer	Shore	12	1.76	0.77
Jun21_S6	Jun_21	Summer	Shore	13	1.82	0.79
Jun21_S7	Jun_21	Summer	Shore	11	1.88	0.81
Jun21_S8	Jun_21	Summer	Shore	14	1.8	0.79
Jun21_S9	Jun_21	Summer	Shore	18	1.24	0.53
Jun21_S10	Jun_21	Summer	Shore	14	1.67	0.75
Jun21_S11	Jun_21	Summer	Shore	16	1.97	0.79
Jun21_S12	Jun_21	Summer	Shore	21	2.11	0.85
Jun21_S13	Jun_21	Summer	Shore	16	1.7	0.69
Jun21_S14	Jun_21	Summer	Shore	17	1.72	0.74
Jun21_S15	Jun_21	Summer	Shore	16	1.57	0.72
Jun21_S16	Jun_21	Summer	Shore	14	1.6	0.73
Jun21_S17	Jun_21	Summer	Shore	16	1.53	0.66
Jun21_S18	Jun_21	Summer	Shore	11	1.55	0.72
Jun21_S19	Jun_21	Summer	Shore	18	1.96	0.81
Jun21_S20	Jun_21	Summer	Shore	20	1.82	0.72
Nov20_M10	Nov_20	Autumn	Midline	6	1.14	0.55
Nov20_M11	Nov_20	Autumn	Midline	5	1.25	0.66
Nov20_M12	Nov_20	Autumn	Midline	10	1.7	0.74
Nov20_M5	Nov_20	Autumn	Midline	19	1.78	0.77
Nov20_N1	Nov_20	Autumn	Nearshore	11	1.89	0.82
Nov20_N2	Nov_20	Autumn	Nearshore	5	1.11	0.59
Nov20_N3	Nov_20	Autumn	Nearshore	14	1.98	0.83
Nov20_N4	Nov_20	Autumn	Nearshore	15	2.06	0.84
Nov20_N5	Nov_20	Autumn	Nearshore	14	2.15	0.86
Nov20_N6	Nov_20	Autumn	Nearshore	15	2.23	0.86
Nov20_N7	Nov_20	Autumn	Nearshore	18	2.24	0.85
Nov20_N8	Nov_20	Autumn	Nearshore	15	2.36	0.89
Nov20_N9	Nov_20	Autumn	Nearshore	8	1.8	0.81
Nov20_S5	Nov_20	Autumn	Shore	13	2.03	0.84
Nov20_S6	Nov_20	Autumn	Shore	12	1.76	0.76
Nov20_S7	Nov_20	Autumn	Shore	14	1.89	0.75
Nov20_S10	Nov_20	Autumn	Shore	13	1.85	0.79

For Review Only

Nov20_S12	Nov_20	Autumn	Shore	11	1.81	0.78
Nov20_S13	Nov_20	Autumn	Shore	15	2.04	0.79
Nov20_S14	Nov_20	Autumn	Shore	14	1.94	0.79
Nov20_S15	Nov_20	Autumn	Shore	17	1.78	0.72
Nov20_S16	Nov_20	Autumn	Shore	15	1.99	0.77
Nov20_S17	Nov_20	Autumn	Shore	11	1.92	0.81
Nov20_S18	Nov_20	Autumn	Shore	11	1.62	0.73
Nov20_S19	Nov_20	Autumn	Shore	16	0.93	0.35
Sep21_M3	Sep_21	Autumn	Midline	16	1.53	0.63
Sep21_M4	Sep_21	Autumn	Midline	16	1.96	0.81
Sep21_M5	Sep_21	Autumn	Midline	19	2	0.76
Sep21_M11	Sep_21	Autumn	Midline	15	1.03	0.39
Sep21_M12	Sep_21	Autumn	Midline	14	1.04	0.55
Sep21_N1	Sep_21	Autumn	Nearshore	13	1.19	0.59
Sep21_N2	Sep_21	Autumn	Nearshore	14	1.18	0.61
Sep21_N4	Sep_21	Autumn	Nearshore	17	2	0.83
Sep21_N5	Sep_21	Autumn	Nearshore	15	1.71	0.71
Sep21_N12	Sep_21	Autumn	Nearshore	21	2.44	0.9
Sep21_S2	Sep_21	Autumn	Shore	17	2	0.81
Sep21_S3	Sep_21	Autumn	Shore	15	1.12	0.45
Sep21_S4	Sep_21	Autumn	Shore	12	2	0.84
Sep21_S5	Sep_21	Autumn	Shore	15	1.95	0.8
Sep21_S6	Sep_21	Autumn	Shore	13	1.52	0.64
Sep21_S7	Sep_21	Autumn	Shore	13	1.45	0.68
Sep21_S9	Sep_21	Autumn	Shore	12	1.5	0.67
Sep21_S10	Sep_21	Autumn	Shore	11	1.81	0.81
Sep21_S17	Sep_21	Autumn	Shore	11	0.81	0.34
Sep21_S18	Sep_21	Autumn	Shore	17	2.06	0.83
Sep21_S20	Sep_21	Autumn	Shore	19	2.17	0.85

For Review Only

Declaration of interests

☐ The authors declare that they have no known competing financial interests or personal relationships that could have appeared to influence the work reported in this paper.

☒ The authors declare the following financial interests/personal relationships which may be considered as potential competing interests:

Zuogang Peng reports financial support was provided by Ministry of Science and Technology of the People's Republic of China. Zuogang Peng reports a relationship with National Natural Science Foundation of China that includes: funding grants. If there are other authors, they declare that they have no known competing financial interests or personal relationships that could have appeared to influence the work reported in this paper.

For Review Only
